# Biome-scale temperature sensitivity of ecosystem respiration revealed by atmospheric CO_2_ observations

**DOI:** 10.1038/s41559-023-02093-x

**Published:** 2023-06-15

**Authors:** Wu Sun, Xiangzhong Luo, Yuanyuan Fang, Yoichi P. Shiga, Yao Zhang, Joshua B. Fisher, Trevor F. Keenan, Anna M. Michalak

**Affiliations:** 1 Department of Global Ecology, Carnegie Institution for Science, Stanford, CA USA; 2 Department of Environmental Science, Policy and Management, University of California, Berkeley, CA USA; 3 Climate and Ecosystem Sciences Division, Lawrence Berkeley National Laboratory, Berkeley, CA USA; 4 Bay Area Air Quality Management District, San Francisco, CA USA; 5 Universities Space Research Association, Mountain View, CA USA; 6 Schmid College of Science and Technology, Chapman University, Orange, CA USA; 7 Present Address: Department of Geography, National University of Singapore, Singapore, Singapore; 8 Present Address: San Francisco, CA USA; 9 Present Address: Sino–French Institute for Earth System Science, College of Urban and Environmental Sciences, Peking University, Beijing, China

**Keywords:** Climate-change ecology, Climate-change impacts

## Abstract

The temperature sensitivity of ecosystem respiration regulates how the terrestrial carbon sink responds to a warming climate but has been difficult to constrain observationally beyond the plot scale. Here we use observations of atmospheric CO_2_ concentrations from a network of towers together with carbon flux estimates from state-of-the-art terrestrial biosphere models to characterize the temperature sensitivity of ecosystem respiration, as represented by the Arrhenius activation energy, over various North American biomes. We infer activation energies of 0.43 eV for North America and 0.38 eV to 0.53 eV for major biomes therein, which are substantially below those reported for plot-scale studies (approximately 0.65 eV). This discrepancy suggests that sparse plot-scale observations do not capture the spatial-scale dependence and biome specificity of the temperature sensitivity. We further show that adjusting the apparent temperature sensitivity in model estimates markedly improves their ability to represent observed atmospheric CO_2_ variability. This study provides observationally constrained estimates of the temperature sensitivity of ecosystem respiration directly at the biome scale and reveals that temperature sensitivities at this scale are lower than those based on earlier plot-scale studies. These findings call for additional work to assess the resilience of large-scale carbon sinks to warming.

## Abstract (web summary)

Atmospheric CO_2_ concentration measured across a network of towers in North America shows that continent- and biome-scale measurements of the temperature sensitivity of ecosystem respiration are lower than have previously been estimated from plot-level studies.

## Main

The terrestrial carbon–climate feedback, resulting from the sensitivity of the terrestrial carbon sink to the physical climate^1^, dominates the uncertainty in climate projections^2–4^. This feedback depends on the difference between the responses of photosynthesis and respiration to a changing climate^5^. Estimates of photosynthesis^6,7^ and respiration^8–10^ vary substantially across terrestrial biosphere models (TBMs)^11^, however. Furthermore, whereas there has recently been a proliferation of novel measurement techniques to better constrain photosynthesis from regional to global scales, such as solar-induced chlorophyll fluorescence^12^, near-infrared reflectance of vegetation^13^ and carbonyl sulfide^14,15^, respiration remains difficult to constrain at large scales due to the absence of a unique spectral signature or atmospheric tracer.

Accurate climate projections thus hinge on improving our understanding of the magnitude, space–time distribution and climatic sensitivity of respiration. More specifically, constraining the temperature sensitivity of respiration is key to estimating total ecosystem respiration, to assessing the extent to which ecosystem respiration will be amplified by a warming climate and to assessing climate-related risks for regions that can trigger a substantial positive carbon–climate feedback^16–19^.

**Extended Data Fig. 1:**
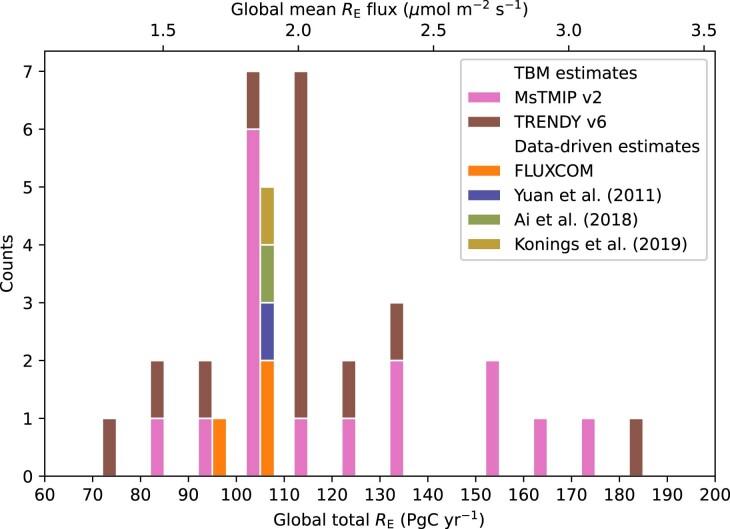
Estimates of global terrestrial ecosystem respiration vary more widely among terrestrial biosphere models than among data-driven models. Terrestrial biosphere model (TBM) estimates of the global total ecosystem respiration (*R*_E_) are shown from the MsTMIP v2 (pink) and TRENDY v6 (brown) model intercomparison projects for the period 2007–2010. Data-driven estimates of global total *R*_E_ include those from three FLUXCOM models (orange) for the same period (2007–2010). In addition, three remote-sensing based estimates from different periods are also included as data-driven estimates, namely, estimates from Yuan et al.^24^ for 2000–-2003 (blue), Ai et al.^25^ for 2001–2010 (green), and Konings et al.^23^ for 2010–2012 (beige). Note that the global estimate from Ai et al.^25^ is rescaled to be consistent with the global domain in Yuan et al.^24^, assuming the same ratio between global *R*_E_ and the total *R*_E_ for land south of 65 ^∘^N and excluding mosaic crops and natural vegetation. The estimate of total ecosystem respiration from Konings et al.^23^ is calculated as the sum of heterotrophic and autotrophic respiration estimates presented in their data product. The top axis shows *R*_E_ in equivalent flux units if averaged over the global land area (1.49 × 10^8^ km^2^).

At regional to global scales, ecosystem respiration (*R*_E_)—the sum of autotrophic and heterotrophic respiratory fluxes—can be estimated from prognostic TBMs that parameterize respiration based on responses to environmental and biotic drivers^20,21^, from data-driven models that rely on site-level measurements^22^, from remotely sensed covariates of respiration^23^ or from a combination thereof^24–26^. TBMs represent respiratory processes in diverse ways^27^ but ultimately rely on generalizing locally derived relationships based on sparsely distributed observations to continental and global scales^28^. This has led to a large spread in estimates of respiration. In addition, estimates of global ecosystem respiration based on TBMs (76–180 Pg C yr^−1^) vary more widely than those based on data-driven models (94–109 Pg C yr^−1^; Extended Data Fig. 1).

Within naturally occurring temperature ranges, ecosystem respiration typically shows an exponential response to temperature^29,30^ as described by the Arrhenius equation^31^ (Methods). While components of ecosystem respiration are also influenced by moisture^32^, phenology^33^, photosynthate input^34,35^, biomass^35^, nutrients^36^, litter quality^37^ and soil microbial responses^38,39^, and especially so at the plot scale and for diurnal to monthly timescales, temperature remains the first-order control of ecosystem respiration (and its primary components) at aggregated spatial (biome to continental) and temporal (monthly to decadal) scales^17,40–43^. This aggregate temperature sensitivity of ecosystem respiration, which incorporates both the direct response of ecosystem respiration to temperature and indirect influences from other climatic and physiological variables, thereby represents the overall response of biome-scale ecosystem respiration to temperature.

Currently, the only available estimates of the biome-scale temperature sensitivity of respiration are from upscaling of plot-scale estimates, which are largely based on observations from eddy covariance towers (for example, Mahecha et al.^44^). Such observations are unevenly distributed across ecosystems^45^ and regions^46,47^ and represent fluxes for areas only up to several km^2^ (refs. ^48–50^). Therefore, even a network of hundreds of sites may not adequately represent fluxes at biome or continental scales^51^. This sampling limitation has led to a large discrepancy between carbon fluxes upscaled from plot-scale observations and those derived from regional-scale observational constraints^26^, which casts doubt on the robustness of using plot-scale estimates to inform certain biome-scale responses. Moreover, cross-site analyses show that temperature sensitivity is similar across ecosystem types^30,44,52^, which contradicts anticipated responses from thermal acclimation of autotrophic and heterotrophic respiration; that is, the warmer the climate, the lower the temperature sensitivity^53–55^. Independent empirical estimates of biome-scale temperature sensitivity of ecosystem respiration, such as those based on regional-scale observational constraints, are thus critically needed.

**Extended Data Fig. 2:**
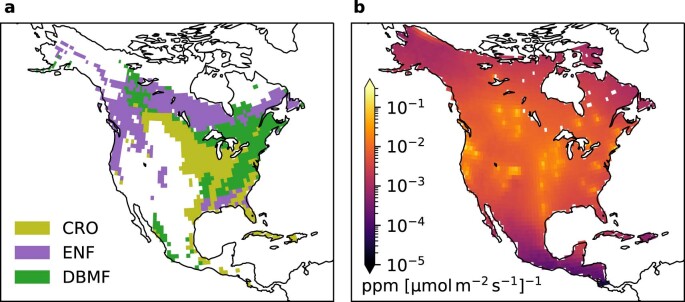
Atmospheric CO_2_ measurements from existing observational networks are most sensitive to fluxes from mesic biomes in temperate and boreal North America. **(a)** The distribution of major biomes in North America: croplands (CRO; olive), evergreen needleleaf forests (ENF; purple), and deciduous broadleaf and mixed forests (DBMF; green). **(b)** Average sensitivity of atmospheric CO_2_ measurements to surface fluxes (ppm [μmol m^−2^ s^−1^]^−1^) over North America during 2007–2010. Hotspots indicate sites of the CO_2_ continuous-monitoring network (Supplementary Table 4). The base map was generated using matplotlib v3.7.1 (https://matplotlib.org) and Cartopy v0.21.1 (https://scitools.org.uk/cartopy).

Here we leverage 39,000 atmospheric CO_2_ concentration measurements from a network of monitoring stations across North America^56^ during the period 2007–2010 to infer temperature sensitivities of ecosystem respiration for North America and major biomes therein (Extended Data Fig. 2a). To do so, we first use model estimates of ecosystem respiration at monthly temporal and 1° × 1° spatial resolution, obtained from the sum of gross primary productivity (GPP) and net ecosystem exchange (NEE, negative for uptake), to evaluate the biome-scale temperature sensitivity of ecosystem respiration as represented by TBM simulations from the Multi-scale Synthesis and Terrestrial Model Intercomparison Project version 2 (MsTMIP v2) (ref. ^57^) and Trends in Net Land–Atmosphere Exchange version 6 (TRENDY v6) (ref. ^58^) ensembles and FLUXCOM machine-learning models^26^. We then assess the degree to which space–time variability in observed atmospheric CO_2_ concentrations is captured by carbon flux estimates from this same ensemble of models. Finally, we use the constraint provided by the atmospheric observations to optimize continental- and biome-scale temperature sensitivities of respiration both for individual models and across the model ensemble.

## Results

### Uncertainty in the temperature sensitivity of respiration

**Fig. 1:**
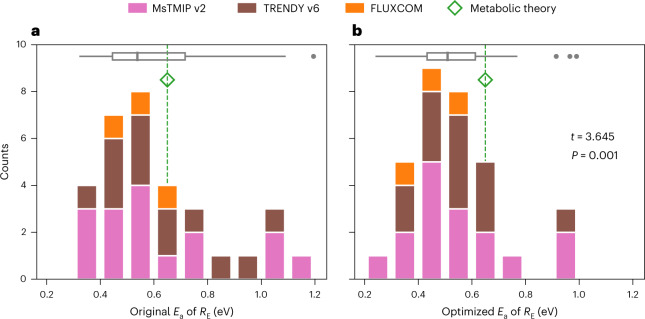
Using atmospheric observations to constrain the temperature sensitivity of respiration for North America reduces the spread in estimates across models and suggests that the large-scale sensitivity is lower than that implied by the metabolic theory of ecology and by plot-scale studies. **a**,**b**, Histograms of the original (**a**) and optimized (**b**) aggregate temperature sensitivity of ecosystem respiration for North America, as represented by *N* = 32 independent estimates of *E*_a_, for TBMs in the MsTMIP v2 (pink) and TRENDY v6 (brown) ensembles and data-driven models in the FLUXCOM ensemble (orange). Grey boxplots summarize the estimates across models, with the centre line, bounds of box, whiskers and dots representing the median, first and third quartiles, smallest and largest estimates falling within 1.5× of the interquartile range from the nearest quartiles and outliers beyond that range, respectively. Green diamonds and vertical dashed lines indicate the reference value of 0.65 eV based on the metabolic theory of ecology^29,59,114^ and plot-scale estimates^52^. A two-tailed, paired two-sample *t* test confirms a statistically significant difference between model-represented temperature sensitivities before (**a**) and after (**b**) optimization against atmospheric CO_2_ observations, as indicated by the *t* statistic and *p* value shown in **b**.

**Extended Data Fig. 3:**
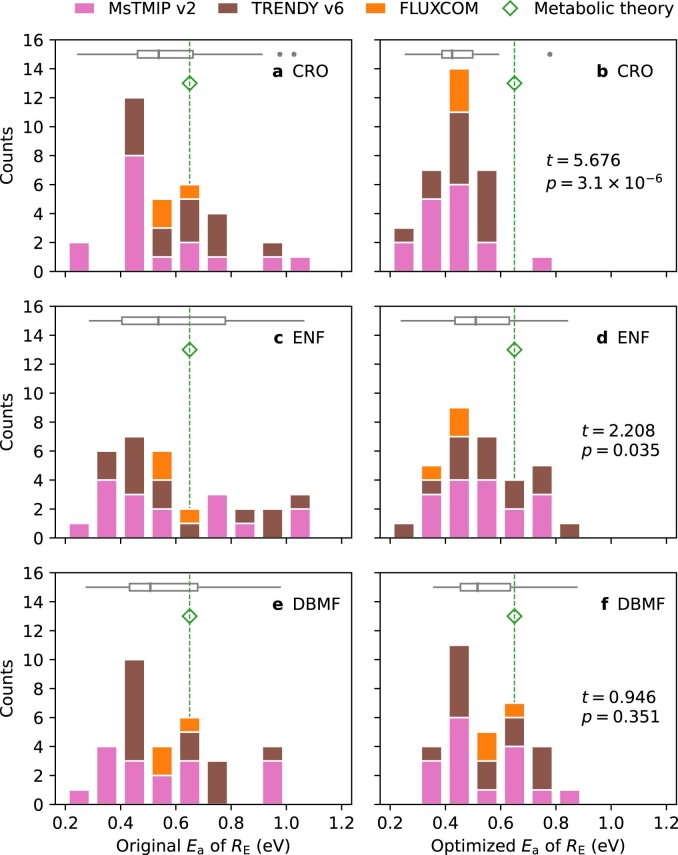
Using atmospheric observations to constrain the temperature sensitivity of respiration for North American biomes reduces the spread in estimates across models and suggests that the large-scale sensitivity is lower than that implied by the metabolic theory of ecology and by plot-scale studies. Similar to Fig. 1, but for individual biomes. Histograms of the original (left) and optimized (right) aggregate temperature sensitivity of ecosystem respiration for croplands (CRO; **a, b**), evergreen needleleaf forests (ENF; **c, d**), and deciduous broadleaf and mixed forests (DBMF; **e, f**), as represented by *N* = 32 independent estimates of the activation energy (*E*_a_), for TBMs in the MsTMIP v2 (pink) and TRENDY v6 (brown) ensembles and data-driven models in the FLUXCOM ensemble (orange). Grey boxplots summarize the estimates across models, with the centre line, bounds of box, whiskers, and dots representing the median, first and third quartiles, smallest and largest estimates falling within 1.5× of the interquartile range from the nearest quartiles, and outliers beyond that range, respectively. Green diamonds and vertical dashed lines indicate the reference value of 0.65 eV based on the metabolic theory of ecology^29,59,114^ and plot-scale estimates^52^. Similar to Fig. 1, *t* statistics and *p* values in (**b, d, f**) indicate differences between model-represented temperature sensitivities before and after optimization against atmospheric CO_2_ observations for CRO, ENF, and DBMF, respectively, according to two-tailed, paired two-sample *t* tests.

**Extended Data Fig. 4:**
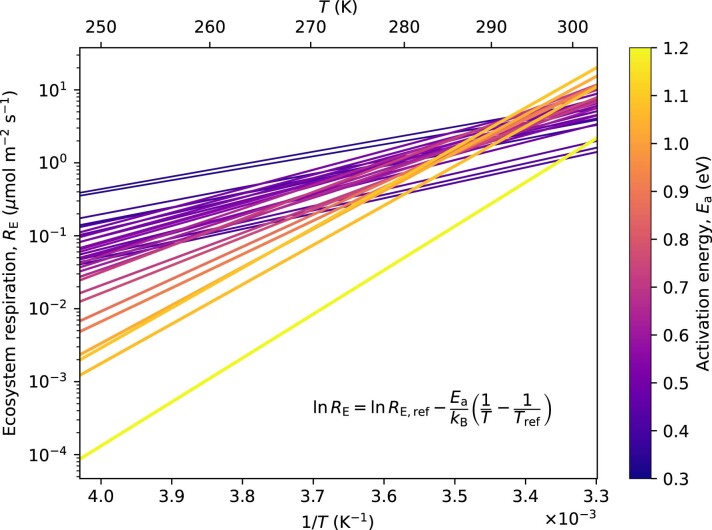
Fitted temperature responses of model estimates of ecosystem respiration. The natural log of ecosystem respiration (*R*_E_) varies linearly with the reciprocal of temperature (1/*T*), according to the Arrhenius equation. Each line is derived from ecosystem respiration estimates from a model simulation in the ensemble and is colour-coded according to the fitted activation energy (*E*_a_, colourbar).

**Extended Data Fig. 5:**
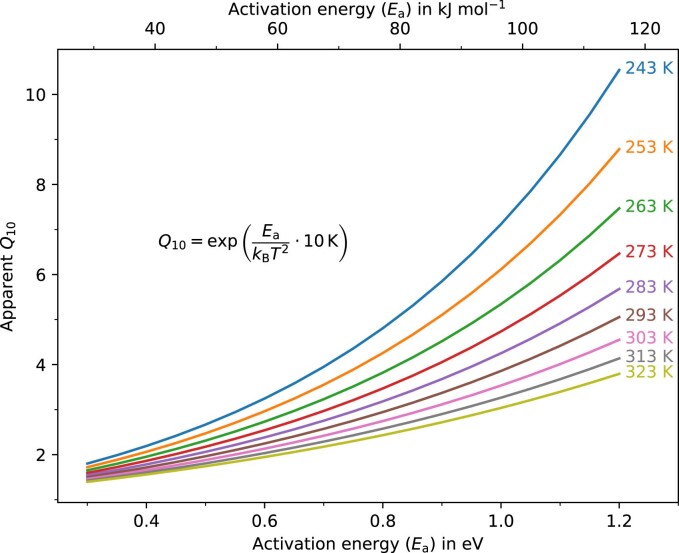
The relationship between the apparent *Q*_10_ and the activation energy (*E*_a_) of a reaction is temperature dependent. For a chemical reaction with a certain activation energy (*E*_a_), *Q*_10_ becomes lower as the temperature increases. See Methods for a derivation of this relationship.

We find that the temperature sensitivity of ecosystem respiration, as represented by the Arrhenius activation energy (*E*_a_, in eV; Methods), ranges widely for the 29 model simulations examined here both for North America (Fig. 1a) and for individual biomes (Extended Data Fig. 3). For North America, for example, *E*_a_ ranges from 0.33 to 1.19 eV (Fig. 1a, Extended Data Fig. 4 and Supplementary Table 1), equivalent to *Q*_10_ values (that is, the factor by which respiration increases for a 10 °C increase in temperature) of 1.5 to 4.9 at 10 °C (Methods and Extended Data Fig. 5). Inferred temperature sensitivities are highly correlated across biomes within the model ensemble; models with a high temperature sensitivity in one biome also tend to have high sensitivities in other biomes (Supplementary Table 2).

Approximately two-thirds of the examined TBM simulations (19 of 29) show an *E*_a_ estimate for North America that is lower than site-level *E*_a_ values derived from flux tower observations^30,52^, which converge around a universal empirical value of *E*_a_ = 0.65 eV for organism- and community-level respiration, according to the metabolic theory of ecology^29,59^ (Fig. 1a). Note that the model-represented, regional-scale estimates derived here are methodologically comparable with previously reported plot-scale temperature sensitivity (0.65 eV) because both are bottom-up estimates and are derived using temperature as the driver, implicitly accounting for the influence of other covariates^30,52^. This discrepancy in temperature sensitivity between regional and plot scales is also present for individual biomes (Extended Data Fig. 3). Interestingly, all three FLUXCOM models, which are trained on site-level flux observations, also exhibit *E*_a_ estimates for North America (0.48 to 0.61 eV; Fig. 1a and Supplementary Table 1) that are lower than those derived directly from half hourly eddy covariance flux tower observations (approximately 0.65 eV (ref. ^52^)), indicating that the upscaling of site-level flux observations in space and time impacts the observed aggregate temperature sensitivity of ecosystem respiration.

These biome-scale aggregate temperature sensitivity estimates may differ from those prescribed in models for individual respiratory components because the former characterize large-scale phenomenological properties resulting from a wide array of underlying microscopic respiratory reactions and encompass mediating effects from GPP and other covariates. For example, the Community Land Model version 4.5 (CLM4.5) prescribes a *Q*_10_ value of 1.5 for autotrophic and heterotrophic respiration^60^, but the activation energy estimated from its model output is 0.62 eV for North America (Supplementary Table 1), equivalent to an apparent *Q*_10_ value of 2.4 at 10°C. On the other hand, it is precisely the fact that these temperature sensitivity estimates reported here characterize the aggregate properties of biomes, which cannot be readily deduced from model parameterizations, that makes them useful for informing the climatic responses of ecosystem respiration and diagnosing implications of model parameterizations.

### Empirical constraint on temperature sensitivities

Next we adjust each model’s activation energy and baseline respiration to maximize consistency with observations of atmospheric CO_2_ concentrations (Methods) and find that the temperature sensitivity of ecosystem respiration is reduced for most (19 of 29) TBMs and all three FLUXCOM models (Fig. 1a,b and Supplementary Table 1). This indicates that the temperature sensitivity across existing prognostic TBMs and data-driven models is higher than what atmospheric observations suggest. The decrease in the optimized temperature sensitivity of ecosystem respiration is especially large for TBMs with high original sensitivity estimates. As a result of the optimization, model spread of the temperature sensitivity of ecosystem respiration also decreases (Fig. 1b), leading to an ensemble mean (± 1 standard error) of 0.54 (± 0.03) eV (and an ensemble median of 0.51 eV). This ensemble mean temperature sensitivity is statistically significantly lower than the previously reported plot-scale temperature sensitivity of 0.65 eV (*p* = 0.00019, one-tailed *Z* test).

**Fig. 2:**
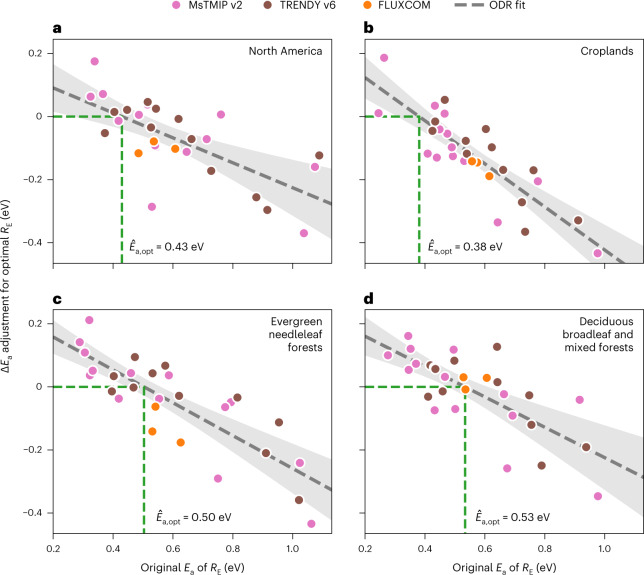
Optimal North American and biome-specific estimates of the temperature sensitivity of ecosystem respiration for individual models and the model ensemble. Adjustments to model-specific estimates of the activation energy are determined by maximizing consistency with observed atmospheric CO_2_ variability (Methods). **a**–**d**, Relationships between adjustments to model-specific estimates of the activation energy needed to maximize consistency with observed atmospheric CO_2_ variability (Δ*E*_a_; vertical axis) and the original estimates of activation energy (*E*_a_; horizontal axis) for models in the MsTMIP v2 (pink), TRENDY v6 (brown) and FLUXCOM (orange) model ensembles in the North American domain (**a**), croplands (**b**), evergreen needleleaf forests (**c**) and deciduous broadleaf and mixed forests (**d**). Note that only models for which the explanatory power of simulated GPP (${R}^{2}_{{{{\rm{GPP}}}}}$) exceeds that of shortwave radiation (${R}^{2}_{{{{\rm{SW}}}}}$) are included (Extended Data Fig. 6), leaving two models in the MsTMIP ensemble and one model in the TRENDY ensemble excluded (Methods). The grey dashed lines represent the best orthogonal distance regression (ODR) fit between Δ*E*_a_ and *E*_a_ estimates across the three ensembles of models (*N* = 29), with light grey shading indicating the 95% prediction interval. The optimal *E*_a_ (${\hat{E}}_{{{{\rm{a,opt}}}}}$) corresponds to the point where the ODR fit line crosses Δ*E*_a_ = 0 eV (that is, no adjustment to *E*_a_ is needed) and is indicated using a green dashed line and listed for each biome in the corresponding panel.

Furthermore, we find that the adjustments to *E*_a_ needed to maximize the consistency with observed atmospheric CO_2_ variability, Δ*E*_a_, are linearly and negatively correlated with the original *E*_a_ estimates of the models (Fig. 2; dashed lines), both for the North American domain (Fig. 2a) and for individual biomes (Fig. 2b–d). This means that atmospheric observations can not only be used to adjust model-specific temperature sensitivities but also to infer an optimal sensitivity (${\hat{E}}_{{{{\rm{a,opt}}}}}$) across models. This ensemble optimal sensitivity is represented by the activation energy corresponding to zero adjustment (that is, Δ*E*_a_ = 0) on the linear relationship between *E*_a_ and Δ*E*_a_.

We find that the best estimate of the temperature sensitivity of ecosystem respiration (${\hat{E}}_{{{{\rm{a,opt}}}}}$) across models is 0.43 ± 0.06 eV (1*σ* uncertainty; equivalent to *Q*_10_ = 1.9 ± 0.2 at 10 °C) for North America. This sensitivity is substantially and significantly lower than the previous global *E*_a_ estimate of 0.65 eV (ref. ^52^) (*p* = 0.00022, one-tailed *Z* test) derived from half hourly, site-level flux observations but is remarkably similar to the *E*_a_ estimate that represents the temperature sensitivity on an annual timescale (0.42 eV (ref. ^52^)). Moreover, the optimal sensitivity (0.43 ± 0.06 eV; Fig. 2a) is also lower than the corrected ensemble mean (0.54 ± 0.03 eV, mean ± 1 s.e.) (*p* = 0.060, one-tailed *Z* test) or median sensitivity (0.51 eV; Fig. 1b) across the models examined here. This difference also shows that absent an observational constraint, the mean or median responses across a model ensemble may not be a good estimate of actual sensitivity.

We also find substantial variability in temperature sensitivity across biomes, namely, 0.38 ± 0.03 eV (*Q*_10_ = 1.7 ± 0.1 at 10 °C) for croplands, 0.50 ± 0.03 eV (*Q*_10_ = 2.1 ± 0.1 at 10 °C) for evergreen needleleaf forests and 0.53 ± 0.05 eV (*Q*_10_ = 2.2 ± 0.1 at 10 °C) for deciduous broadleaf and mixed forests, which contrasts with earlier site-level studies that had suggested that *E*_a_ is uniform across a range of ecosystem types^30,52^. Tropical and Arctic biomes are not considered individually here due to the low sensitivities of available atmospheric CO_2_ observations to surface fluxes in these regions (Extended Data Fig. 2b).

Additional analyses confirm that the temperature sensitivities inferred here are not substantially impacted by potential confounding effects of soil moisture or radiation, by co-variability with GPP, by thermal acclimation or by lateral fluxes (Supplementary Notes 1–5 and Supplementary Figs. 1–7). These inferred temperature sensitivities therefore do represent the overall biome-scale response of ecosystem respiration to climatic temperature gradients.

### Correcting temperature sensitivity improves model NEE skill

Having obtained observationally constrained estimates of temperature sensitivities of ecosystem respiration (Fig. 2), we then examine how these estimates impact the ability of NEE estimates from TBMs and data-driven models to explain observed atmospheric CO_2_ variability.

**Fig. 3:**
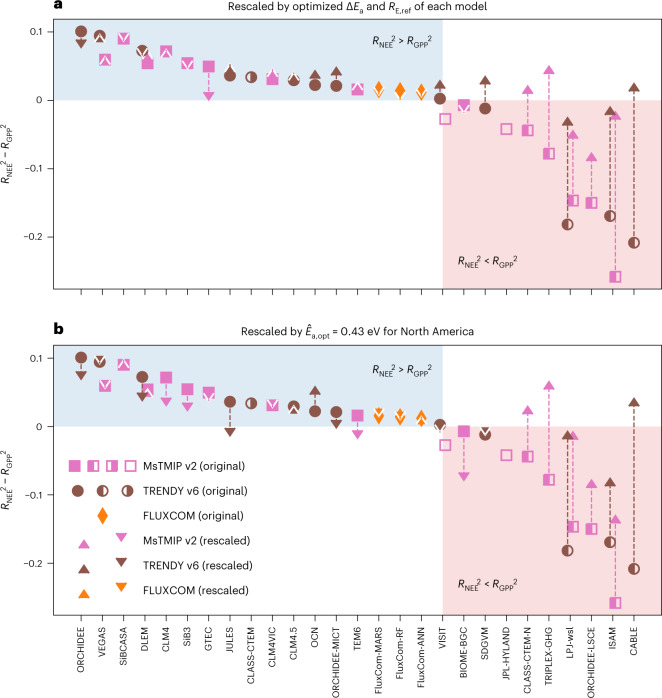
Rescaling ecosystem respiration by adjusting *E*_a_ to optimized values leads to substantial improvement in NEE explanatory power for models for which the explanatory power of NEE originally trailed that of GPP. **a**, The difference between the explanatory power of NEE (${R}^{2}_{{{{\rm{NEE}}}}}$) and GPP (${R}^{2}_{{{{\rm{GPP}}}}}$) before and after rescaling based on model-specific optimal adjustments to the temperature sensitivity (Δ*E*_a_ in Fig. 2a) and the baseline respiration rate at 10^∘^C for North America (Extended Data Fig. 7a). **b**, The same information from **a** after rescaling based on an overall optimal temperature sensitivity derived for North America (${\hat{E}}_{{{{\rm{a,opt}}}}}=0.43$ eV; Fig. 2a). The *R*^2^ differences (${R}^{2}_{{{{\rm{NEE}}}}}-{R}^{2}_{{{{\rm{GPP}}}}}$) of the original MsTMIP, TRENDY and FLUXCOM models are represented by squares, circles and diamonds, respectively. A symbol that is filled in the left (right) half indicates that the corresponding model’s GPP (NEE) estimates explain a higher fraction of CO_2_ variability than does incoming shortwave radiation (${R}^{2}_{{{{\rm{SW}}}}}=0.23$). Solid symbols indicate models for which both GPP and NEE estimates outperform shortwave radiation in explaining atmospheric CO_2_ variability, whereas empty symbols indicate models for which neither GPP nor NEE estimates outperform shortwave radiation. Models for which the explanatory power of GPP lags behind that of shortwave radiation (CLASS-CTEM, VISIT and JPL-HYLAND) are excluded from the rescaling. The blue shaded area highlights models for which the original estimates of NEE outperform GPP in terms of explanatory power, while the red shaded area includes models for which GPP outperforms NEE. The *R*^2^ differences after rescaling respiration are indicated by triangles, with upward orientation indicating an improvement in the explanatory power and downward orientation indicating the opposite. Most models for which the explanatory of NEE originally lagged that of GPP (red shaded regions of both panels) show a marked improvement in explanatory power once the temperature sensitivity of ecosystem respiration is adjusted to optimized values. Conversely, adjusting the temperature sensitivity of respiration does not have a large or consistent impact for those models where the explanatory power of NEE was already superior to that of GPP (blue shaded regions). See Supplementary Table 3 for a list of all model names, abbreviations and references.

**Extended Data Fig. 6:**
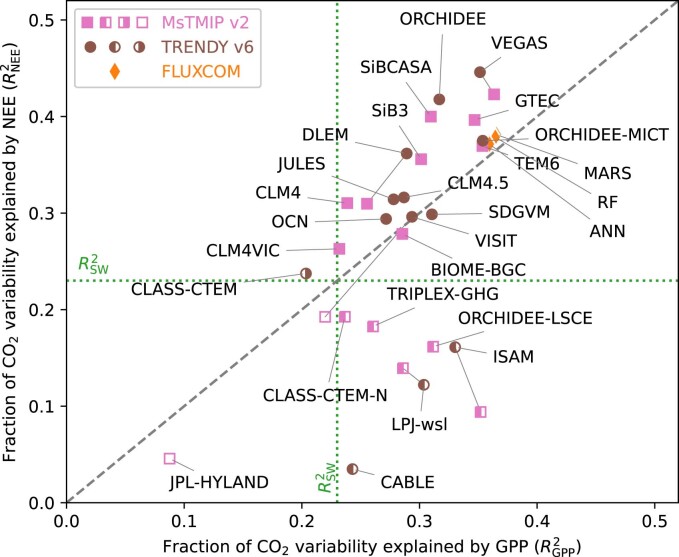
For some models, NEE estimates do not outperform the same models’ GPP estimates in explaining observed atmospheric CO_2_ variability. Fractions of observed CO_2_ variability explained by NEE estimates (${R}^{2}_{{{{\rm{NEE}}}}}$) vs. those explained by GPP estimates (${R}^{2}_{{{{\rm{GPP}}}}}$) from simulations from the MsTMIP v2 (pink squares), TRENDY v6 (brown circles), and FLUXCOM (orange diamonds) model ensembles for 2007–2010. A model that falls below the one-to-one dashed line has GPP estimates that explain observed atmospheric CO_2_ variability better than that same model’s NEE estimates. Similar to Fig. 3, symbols that are half filled to the left (right) indicate models whose GPP (NEE) estimates explain a higher fraction of CO_2_ variability than does incoming shortwave radiation (${R}^{2}_{{{{\rm{SW}}}}}=0.23$; green dotted line). Data points that represent the same model participating in different ensembles (for example, LPJ-wsl in MsTMIP and TRENDY) are linked with thin lines.

Surprisingly, although space–time variability in atmospheric CO_2_ concentrations results from space–time patterns in NEE, we find that GPP estimates explain the variability in observed atmospheric CO_2_ concentrations better than corresponding NEE estimates for 12 of the 29 original TBM simulations in the MsTMIP and TRENDY ensembles (Fig. 3; circles, squares or diamonds; Extended Data Fig. 6). The difference between the explanatory power of monthly averaged NEE estimates and that of GPP estimates (${{\Delta }}{R}^{2}={R}^{2}_{{{{\rm{NEE}}}}}-{R}^{2}_{{{{\rm{GPP}}}}}$) ranges from Δ*R*^2^ = 0.10 for the simulation for which the improvement from GPP to NEE is greatest to Δ*R*^2^ = −0.26 for the simulation where the deterioration relative to the explanatory power of GPP is greatest, highlighting the misrepresentation of ecosystem respiration in the latter set of models. For the FLUXCOM models (not considered TBMs because of their data-driven nature; Methods), NEE estimates neither markedly improve nor degrade the degree to which flux estimates reproduce observed atmospheric CO_2_ variability relative to GPP estimates (Δ*R*^2^ ≤ 0.02; Fig. 3). Note that three of the 29 TBM simulations have GPP estimates that explain an even smaller portion of the observed atmospheric CO_2_ variability than does shortwave radiation (${R}^{2}_{{{{\rm{SW}}}}}=0.23$; symbols with empty left portion in Fig. 3; Extended Data Fig. 6), a first-order climatic driver of GPP, and these simulations are therefore excluded from further analysis (consistent with Fig. 2; Methods).

**Extended Data Fig. 7:**
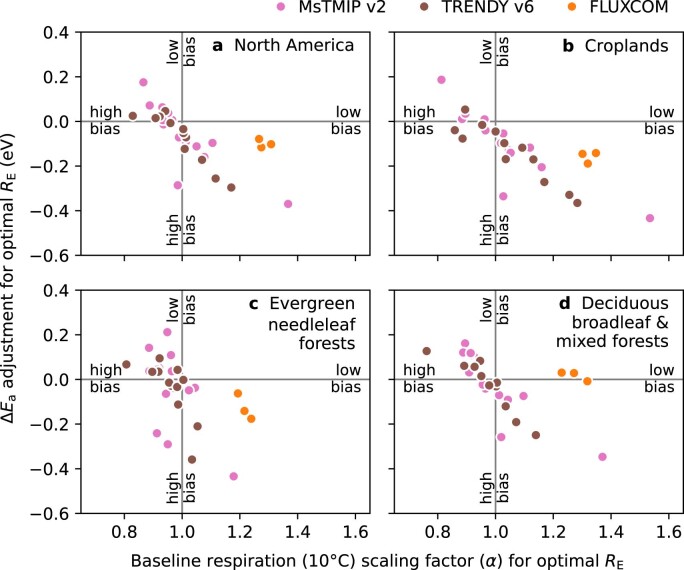
Attribution of respiration bias to the temperature sensitivity and the baseline respiration rate based on maximal consistency with atmospheric CO_2_ observations for (a) the North American domain, (b) croplands, (c) evergreen needleleaf forests, and (d) deciduous broadleaf and mixed forests. TBMs and the FLUXCOM models are colour-coded circles, as in the legend. Note that a negative Δ*E*_a_ adjustment means that the original *E*_a_ parameter of the model has a high bias, and a positive Δ*E*_a_ adjustment means a low bias in *E*_a_. Similarly, for the baseline respiration adjustment, a ratio greater than one indicates a low bias in the original baseline respiration of the model, and a ratio smaller than one indicates a high bias in baseline respiration. For most TBMs, a low bias in *E*_a_ is compensated by a high bias in the baseline respiration (the 2nd quadrant), or a high bias in *E*_a_ is compensated by a low bias in the baseline respiration (the 4th quadrant). However, FLUXCOM models tend to show a low bias in the baseline respiration and a small to no bias in temperature sensitivity.

Correcting ecosystem respiration by adjusting temperature sensitivity and baseline respiration (Extended Data Fig. 7 and Methods) substantially improves the degree to which NEE estimates reproduce observed atmospheric CO_2_ variability for those models for which the performance of NEE trailed that of GPP (Fig. 3a, red shaded area). For four of the ten models in this group (SDGVM, CLASS-CTEM-N, CABLE and TRIPLEX-GHG; Supplementary Table 3), NEE estimates in fact outperform GPP estimates once ecosystem respiration is corrected. Only one model, BIOME-BGC (Supplementary Table 3), which has the lowest temperature sensitivity among all investigated models (*E*_a_ = 0.33 eV; Supplementary Table 1), shows a minor degradation after respiration is corrected.

By contrast, correcting ecosystem respiration does not consistently improve the performance of NEE estimates for models for which the explanatory power of NEE already exceeded that of GPP (Fig. 3a, blue shaded area). For these models, bias in ecosystem respiration may have been offset by corresponding bias in GPP.

Interestingly, correcting ecosystem respiration using a single value of the ensemble optimal temperature sensitivity (0.43 eV for North America) across all models and biomes yields an improvement in the performance of models’ estimates of NEE that is almost as large (mean increase in ${R}^{2}_{{{{\rm{NEE}}}}}$ of 0.10 for models for which ${R}^{2}_{{{{\rm{NEE}}}}} < {R}^{2}_{{{{\rm{GPP}}}}}$; Fig. 3b) as adjusting both the respiration sensitivity and baseline respiration to model-specific optimized values (mean increase in ${R}^{2}_{{{{\rm{NEE}}}}}$ of 0.12 for the same set of models; Fig. 3a), further supporting the robustness of the overall estimate of temperature sensitivity for North America.

The improvement in model explanatory power of NEE after correcting ecosystem respiration indicates a dominant role of the temperature sensitivity bias in causing the underperformance in carbon flux estimates, although other sources of bias also exist. Attribution of model NEE bias to respiration parameters has been difficult up to now because of equifinality—that is, in the presence of respiration bias, the bias in NEE estimates may still be alleviated by compensation from bias in GPP estimates. Here because rescaling ecosystem respiration based on an overall optimal temperature sensitivity for North America alone (Fig. 3b; Methods) leads to substantial improvement in NEE explanatory power for models for which the performance of NEE trails that of GPP (Δ*R*^2^ < 0; Fig. 3b, red shaded area), respiration bias is probably a more important contributor to the NEE bias than is GPP bias for these models. This notion is also corroborated by changes in the seasonal cycles of NEE after imposing the optimal temperature sensitivity on the model ensemble (Supplementary Figs. 8 and 9) and by additional tests on the influences of GPP uncertainty on the inferred temperature sensitivity (Supplementary Figs. 1 and 2).

## Discussion

Our findings highlight the spatial-scale dependence of the temperature sensitivity of respiration. Several factors probably contribute to the temperature sensitivity at the biome scale and seasonal to interannual timescales being lower than that reported from plot-scale studies. Temporal aggregation may smooth out short-term (for example, half hourly, typical for plot-scale observations) responses, leading to markedly reduced temperature sensitivity on annual timescales^30,52^. For soil heterotrophic respiration—a major component of ecosystem respiration—climatological temperature sensitivity has also been shown to differ from instantaneous temperature sensitivity^17^. Indeed, we find that the relationship between biome-scale temperature sensitivity estimates and mean air temperatures of the studied biomes (Supplementary Fig. 10) is broadly consistent with the previously observed response of climatological *Q*_10_ for soil carbon decomposition to the mean air temperature in the temperate domain^17^. Despite the difference in respiratory components, such qualitative consistency lends support to the robustness of the inferred climatologically relevant temperature sensitivity for temperate North American biomes.

Moreover, biome-scale temperature sensitivities may further differ from plot-scale temperature sensitivities because the responses observed here incorporate indirect sensitivities to temperature via drivers such as soil moisture^32^, nutrients^36^ and phenology^33^. Indeed, some plot-scale studies show a lower temperature sensitivity than the biome-scale temperature sensitivity inferred here after removing influences from low-frequency variability (*Q*_10_ = 1.4 ± 0.1 (ref. ^44^)) or hydrometeorological drivers (*Q*_10_ = 1.6 ± 0.1 (ref. ^61^)). In addition, as ecosystems in cooler (warmer) climates tend to have higher (lower) baseline respiration rates^29^, accounting for this spatial gradient in baseline respiration potentially dampens the inferred biome-scale temperature sensitivity. Ecosystem state variables such as GPP and phenology may also regulate the baseline respiration^62^. The relative contribution of these factors at the biome scale merits further research. Given that the sensitivities inferred here encompass these various additional factors, they are likely to more aptly reflect the bulk response of respiration to future warming on aggregate space–time scales and therefore inform climatic responses.

In addition to differences in sensitivity across scales, several known challenges with flux measurements and partitioning may have also led to an overestimate of the temperature sensitivity of ecosystem respiration in earlier studies even at the plot scale. These known challenges can cause ecosystem respiration to be undercounted when temperatures are low (at night or in the dormant season) and overestimated when temperatures are high (during the day or in the growing season). Nighttime respiration can be underestimated in conditions of weak turbulence and canopy CO_2_ storage build-up^63^. Moreover, for sites in uneven terrain, there is no accepted way to reliably account for advective CO_2_ fluxes^64^, which may lead to a further underestimation of respiratory carbon loss at night. Partitioning daytime respiration from NEE measurements also remains challenging because the relationship between nighttime respiration and temperature often does not hold during the daytime due to light inhibition of leaf respiration^65–67^ and non-temperature controls of nighttime autotrophic respiration^68^. As a result, using the relationship between nighttime respiration and temperature to partition daytime fluxes often leads to overestimation of daytime respiration^69^. Furthermore, this overestimation of daytime respiration is particularly strong in the growing season (up to 23%) (ref. ^69^), further enhancing the bias of inferred temperature sensitivity on an annual timescale. These problems all contribute to a potential positive bias in the plot-scale temperature sensitivity and, in turn, the size of the difference in temperature sensitivity across scales reported here.

The difference in the temperature sensitivity of ecosystem respiration between croplands and forests observed here (Fig. 2b-d) and the absence of such biome specificity in plot-scale studies may arise from several factors. Croplands typically show a higher carbon use efficiency (ratio between net primary productivity and GPP) than unmanaged forests^70^, that is, a lower autotrophic fraction in ecosystem respiration. This, in turn, means that the fraction of soil heterotrophic respiration, which is less sensitive to air temperature than is aboveground respiration, is higher in croplands than forests, thereby leading to the overall lower temperature sensitivity of ecosystem respiration in croplands. Management practices such as harvest, irrigation and tillage can cause dramatic changes in ecosystem respiration at weekly to monthly timescales^71^, though their impact on the temperature sensitivity at longer timescales remains poorly understood. The dearth of cropland sites in FLUXNET observations^45^ may explain the lack of biome specificity in the temperature sensitivity inferred in plot-scale studies and the fact that the FLUXCOM models^26^, trained on FLUXNET observations, capture the temperature sensitivity best in deciduous broadleaf and mixed forests (Fig. 2d) but least well in croplands (Fig. 2b). To reach a robust understanding of cropland carbon cycling, future investigations may need to examine the partitioning among different respiratory components, quantify the impact of management practices on the temperature sensitivity of ecosystem respiration and expand the eddy covariance network in cropland areas.

Given that the temperature sensitivity of ecosystem respiration constitutes a leading-order positive climate feedback, the findings presented here also open several avenues for advancing the understanding of terrestrial carbon–climate feedbacks.

First, although observed atmospheric CO_2_ variability incorporates influences of both photosynthesis and ecosystem respiration, the inference of an ensemble optimal temperature sensitivity for respiration for North America (Fig. 2a) and major biomes (Fig. 2b–d) indicates that the current level of uncertainty in GPP^7,72,73^ does not obscure information about ecosystem respiration (Supplementary Fig. 1). Given this finding, partitioning of net carbon fluxes into photosynthesis and respiration at regional scales is, in principle, achievable in a manner akin to the respiration-based partitioning widely used at eddy covariance sites^63,74^; such partitioning would overcome a key observational challenge in constraining regional-scale responses of carbon fluxes to climate. Pursuing this goal may require expanding and optimizing atmospheric observational networks to better resolve space–time variability in photosynthesis and respiration^75^, especially in sparsely sampled tropical and Arctic biomes.

Second, our findings establish a link between the explanatory power of modelled NEE and bias in the temperature sensitivity of ecosystem respiration, which is useful for refining TBMs. Although the temperature sensitivity of either GPP or ecosystem respiration may be adjusted to improve the consistency between NEE and observed atmospheric CO_2_ concentrations, in reality, GPP and ecosystem respiration are intricately tied by carbon allocation and decomposition processes. Such coupling does not always allow errors in ecosystem respiration to be cancelled by corresponding errors in GPP. Here the substantial improvements in model explanatory power resulting from correcting the temperature sensitivity of ecosystem respiration (Fig. 3) provide robust evidence that current model representation of respiratory processes contribute substantially to uncertainty in NEE estimates, and in turn, uncertainty in the response of the terrestrial carbon sink to future warming. We suggest that calibration of the biome-scale temperature sensitivity of ecosystem respiration against multi-scale constraints be prioritized in model development to reduce compensating errors in ecosystem respiration and GPP, which could otherwise meet model benchmarks but for incorrect reasons.

Finally, if bias in the temperature sensitivity of ecosystem respiration represented by standalone TBM simulations is indicative of that in coupled Earth system model simulations, we may expect the response of ecosystem respiration to warming to be stronger in those models than what current atmospheric CO_2_ observations suggest as well. This difference also highlights a gap in the predictive understanding of how large-scale carbon sinks respond to future warming, because much of current understanding derives from Earth system model simulations. Meanwhile, in the long run, the response of ecosystem respiration to climatic warming may be additionally influenced by acclimation to a warmer climate^53–55,76,77^, changes in soil microbial community composition^78^, limitation of labile carbon pools^79^ and soil warming^80^, among other factors. As early warning signs of potential saturation and destabilization of regional carbon sinks appear^81^, there is an urgent need to assess the resilience of large-scale carbon sinks to climatic warming by synthesizing multi-scale observational constraints with Earth system models that embed state-of-the-art mechanistic understanding of respiration. Looking forward, we anticipate that the expanding network of ground-based, airborne and satellite remote-sensing observations of atmospheric CO_2_ concentrations will further elucidate the climatic sensitivities of ecosystem respiration across regions and spatiotemporal scales and thereby inform respiration-driven terrestrial carbon–climate feedbacks.

## Methods

### Model estimates of terrestrial carbon fluxes

Monthly model estimates of GPP and NEE were obtained from the MsTMIP (version 2, spanning 1901–2010)^57,82–86^ and the TRENDY (version 6, spanning 1960–2016)^58,87,88^ ensembles for the period 2007–2010. We chose this study period because of the overlapping temporal coverage of carbon flux estimates (MsTMIP ends in 2010) and high-resolution atmospheric transport (starting in 2007). Each model ensemble used a set of standardized climate drivers to drive individual model runs, thereby minimizing model divergence contributed from climate drivers. There were 29 simulations from 24 independent TBMs. In addition, we used three machine-learning models from the FLUXCOM ensemble for comparison^26^. This yielded *N* = 32 pairs of simulated GPP and NEE estimates for the analysis of ecosystem respiration. The model output was regridded to 1° × 1° resolution. A list of all models is provided in Supplementary Table 3.

### Estimating the temperature sensitivity of respiration

Air temperature data were obtained from the North American Regional Reanalysis data^89^, regridded to the same monthly, 1° × 1° resolution as the carbon flux estimates.

Ecosystem respiration (*R*_E_) was calculated from the sum of model estimates of GPP and NEE per definition (note that NEE is negative when there is net ecosystem uptake).

We used the Arrhenius equation to describe the relationship between ecosystem respiration (*R*_E_, μmol m^−2^ s^−1^) and air temperature (*T*, K):

1 $${R}_{{{{\rm{E}}}}}(T)={R}_{{{{\rm{E,ref}}}}} \exp \left[-\frac{{E}_{{{{\rm{a}}}}}}{{k}_{{{{\rm{B}}}}}}\left(\frac{1}{T}-\frac{1}{{T}_{{{{\rm{ref}}}}}}\right)\right],$$

where *E*_a_ (eV) is the activation energy of ecosystem respiration, *k*_B_ = 8.617333262 × 10^−5^ eV K^−1^ is the Boltzmann constant, *T*_ref_ = 10 °C or 283.15 K is the reference temperature and *R*_E,ref_ (μmol m^−2^ s^−1^) is the baseline ecosystem respiration rate at *T*_ref_.

We estimated *E*_a_ and *R*_E,ref_ for each model for North America (Fig. 1a) and then also separately for several major biomes (croplands, evergreen needleleaf forests and deciduous broadleaf and mixed forests; Extended Data Fig. 2a) through a linear regression:

2 $$\ln {R}_{{{{\rm{E}}}}}(T)=\ln {R}_{{{{\rm{E,ref}}}}}-\frac{{E}_{{{{\rm{a}}}}}}{{k}_{{{{\rm{B}}}}}}\left(\frac{1}{T}-\frac{1}{{T}_{{{{\rm{ref}}}}}}\right).$$

*R*_E_ values that were negative or close to zero were filtered out. We used all valid monthly, grid-cell-level *R*_E_ and corresponding temperature values during the period 2007–2010 to estimate *E*_a_ and *R*_E,ref_ for each study domain (North America or individual biomes), accounting for both spatial and temporal variabilities in temperature and *R*_E_. This treatment took the assumption of ‘trading space for time’^54,90^, given the short span of the study period. Note that we did not consider temperature thresholds in the response of ecosystem respiration^52^ because monthly mean temperature rarely dips below the previously identified low temperature threshold (−24.8 °C) and most models do not show a discontinuity above the high temperature threshold (15.1 °C) in the study domain.

The *Q*_10_ formulation expresses respiration as an exponential function of temperature, that is, a linear relationship between $\ln {R}_{{{{\rm{E}}}}}$ and *T*. Hence we linearized $\ln {R}_{{{{\rm{E}}}}}$ against *T* to obtain the apparent *Q*_10_ as a function of *E*_a_ and *T* to aid interpretation of estimated *E*_a_ values in the context of reported *Q*_10_ values in the literature:

3 $${Q}_{10}\left({E}_{{{{\rm{a}}}}},T\right)=\exp \left(\frac{\partial \ln {R}_{{{{\rm{E}}}}}}{\partial T} {\Delta T}_{10}\right)=\exp \left(\frac{{E}_{{{{\rm{a}}}}}}{{k}_{{{{\rm{B}}}}}{T}^{2}}{\Delta T}_{10}\right),$$

where Δ*T*_10_ = 10 K. For example, at *T* = 283.15 K (10 °C), *E*_a_ = 0.65 eV is equivalent to an apparent *Q*_10_ of 2.6 (Extended Data Fig. 5).

### Optimizing the temperature sensitivity of respiration

Observations of atmospheric CO_2_ concentrations are sensitive to net CO_2_ fluxes from large regions (10^6^ km^2^) (ref. ^91^) and thus provide top-down constraints on carbon flux estimates for large biomes and continental regions^73,92–96^, provided that atmospheric transport is adequately resolved to link fluxes to observed concentrations. While atmospheric CO_2_ observations can inform the mass balance and space–time variability of net carbon fluxes, they lack direct component-level constraints on photosynthesis and respiration. Consequently, TBM-based estimates^97^ or photosynthetic proxies^9,98^ have been needed to separately inform estimates of photosynthesis and respiration from atmospheric CO_2_ observations.

Here we optimized the continental- and biome-scale temperature sensitivity of ecosystem respiration for individual models by maximizing the consistency between transported signals of ecosystem respiration from bottom-up models and the components of space–time variability in CO_2_ concentrations caused by ecosystem respiration (Extended Data Fig. 2).

To do so, atmospheric measurements of CO_2_ mixing ratio during 2007–2010 were obtained from 44 tall towers across North America, sourced from the ObsPack CO_2_ GlobalViewPlus v3.2 data product^56,99^. We used three hourly averaged CO_2_ measurements centred in the afternoon (15:00 local time for most sites and 16:00 or 17:00 for a few remaining sites). From these measurements, we subtracted background CO_2_ values and fossil fuel influences (FFDAS v2 (Fossil Fuel Data Assimilation System version 2)) (ref. ^100^) to derive the net influence of terrestrial biospheric carbon fluxes on atmospheric CO_2_ mixing ratio. The resulting observations of CO_2_ depletion or enhancement were then filtered to remove influences from non-terrestrial fluxes or data points that showed a large model–data mismatch. In sensitivity analyses, we also subtracted the influence of lateral fluxes obtained from the Global Stocktake data product^101–104^ (Supplementary Notes 2). We then removed the influence of GPP on atmospheric CO_2_ observations in the same way. This yielded the respiratory component of the space–time variability in atmospheric CO_2_ observations. Because biomass-burning emissions (~0.06 Pg C yr^−1^) (ref. ^105^) were minor compared with NEE over North America (−0.7 Pg C yr^−1^) (ref. ^95^) during the study period (2007–2010)—and certainly dwarfed by model spread in ecosystem respiration—influences from biomass-burning emissions on the temperature sensitivity of ecosystem respiration were not considered.

Sensitivities of CO_2_ mixing ratio measurements to surface fluxes, also known as transport footprints, were produced from high-resolution (10 km for temperate North America and 40 km for tropical and high-latitude North America) WRF–STILT (Weather Research and Forecasting – Stochastic Time Inverted Lagrangian Transport) model runs^106^ and post aggregated to a 1° × 1° resolution as part of the CarbonTracker–Lagrange project (https://gml.noaa.gov/ccgg/carbontracker-lagrange/). The potential transport error is demonstrably minor relative to the spread in TBM flux estimates^107,108^.

We then calculated the adjustments to the temperature sensitivity (*E*_a_) and the baseline respiration rate (*R*_E,ref_) by minimizing the mismatch between the transported signal of model estimates of ecosystem respiration and the respiratory component of atmospheric CO_2_ variability. The original estimates of ecosystem respiration (*R*_E_, equation (1)) were adjusted as follows:

4 $${R}_{{{{\rm{E}}}}}\!^{* }={R}_{{{{\rm{E}}}}} \alpha \exp \left[-\frac{{{\Delta }}{E}_{{{{\rm{a}}}}}}{{k}_{{{{\rm{B}}}}}}\left(\frac{1}{T}-\frac{1}{{T}_{{{{\rm{ref}}}}}}\right)\right],$$

where ${R}_{{{{\rm{E}}}}}\!^{* }$ (μmol m^−2^ s^−1^) is the adjusted estimate of ecosystem respiration, Δ*E*_a_ (eV) is the adjustment to *E*_a_ needed to minimize the mismatch and *α* > 0 is a dimensionless scaling factor for the baseline respiration rate.

Like the original estimates of *E*_a_, the adjustments Δ*E*_a_ were estimated over the North American domain and for major biomes. The parameter *α* accounted for the adjustment to the magnitude of ecosystem respiration (Extended Data Fig. 7), thereby allowing the bias from temperature sensitivity and that from the seasonal cycle amplitude to be separately constrained.

To obtain an estimate of the overall optimal *E*_a_ for North America (and for individual biomes) across models, we fitted linear relationships between Δ*E*_a_ adjustments and the original *E*_a_ estimates for individual models using the orthogonal distance regression. Orthogonal distance regression was used because it accounts for errors in both the regressor and the response^109^. The fitting was performed using the SciPy ODR function^110,111^. An optimal value of *E*_a_, that is, ${\hat{E}}_{{{{\rm{a,opt}}}}}$ was derived at the intersection of Δ*E*_a_ = 0 and the best fit line for each domain (North America or individual biomes; Fig. 2).

### Evaluating carbon flux estimates using observations

We used in situ measurements of atmospheric CO_2_ mixing ratio and the transport footprints to determine the extent to which regional-scale estimates of GPP and NEE are consistent with observations. As in Sun et al.^73^, we calculated the transported signal of GPP or NEE estimates for each individual model and assessed how well the transported signal explains the biospheric component of the observed CO_2_ space–time variability using the coefficient of determination (*R*^2^).

We used the explanatory power of shortwave radiation, ${R}^{2}_{{{{\rm{SW}}}}}=0.23$, as a minimum benchmark to exclude a small subset of models with very low explanatory power (*N* = 3) from the subsequent analysis on ecosystem respiration. Shortwave radiation data were obtained from the North American Regional Reanalysis data^89^. Because shortwave radiation is a first-order climatic driver of GPP^112^ and influential in determining NEE space–time variability^113^, we would expect carbon flux estimates to explain at least as much portion of observed atmospheric CO_2_ variability as the shortwave radiation.

### Evaluating temperature sensitivity-corrected NEE estimates

We assessed how updating the temperature sensitivity of respiration (‘Optimizing the temperature sensitivity of respiration’) impacted the explanatory power of NEE estimates.

To do so, we adjusted ecosystem respiration estimates for each model according to the optimal Δ*E*_a_ and *α* derived for that model in the optimization procedure (equation (4)). Note that correction to the baseline respiration rate through *α* changes the mean ecosystem respiration magnitude (equation (4)), which avoids erroneously adjusting the temperature sensitivity in response to a bias in overall magnitude.

In a second analysis, we instead adjusted *E*_a_ of all models to the optimal value over North America (${\hat{E}}_{{{{\rm{a,opt}}}}}=0.43$ eV) estimated from the model ensemble. The adjustment of *R*_E_ was conducted by multiplying *R*_E_ by the temperature sensitivity adjustment, $\exp (-\frac{{{\Delta }}{E}_{{{{\rm{a}}}}}}{{k}_{{{{\rm{B}}}}}T})$, followed by rescaling the magnitude to conserve the mean *R*_E_. We also capped the adjusted *R*_E_ at its original maximum value $\max ({R}_{{{{\rm{E}}}}})$ within a relative tolerance level (≤1%) to prevent unrealistically high values being produced by the exponential function.

The performance of the two sets of adjusted NEE estimates in explaining the space–time variability in atmospheric CO_2_ measurements was evaluated using the same approach as described in the previous section.

### Reporting summary

Further information on research design is available in the Nature Portfolio Reporting Summary linked to this article.

## Supplementary information

Supplementary Information Supplementary Tables 1–4, Figs. 1–10 and Notes 1–6.

Reporting Summary

## Data Availability

Results presented in this study are available at 10.5281/zenodo.7874439. The ObsPack GLOBALVIEWplus CO_2_ data product is available at https://www.esrl.noaa.gov/gmd/ccgg/obspack. The CarbonTracker–Lagrange WRF–STILT footprints are available at https://gml.noaa.gov/ccgg/carbontracker-lagrange/. The FFDAS v2 data product is available at https://ffdas.rc.nau.edu/. The North American Regional Reanalysis data can be obtained at https://psl.noaa.gov/data/gridded/data.narr.html. The MsTMIP v2 model ensemble is available at https://nacp.ornl.gov/. The TRENDY v6 model ensemble is available at https://blogs.exeter.ac.uk/trendy/. The FLUXCOM model ensemble is available at http://www.fluxcom.org/.

## Code availability

Code used to generate figures and results is available at 10.5281/zenodo.7874439.
