# Supplementary material for: Biome-scale temperature sensitivity of ecosystem respiration revealed by atmospheric CO2 observations
Source: Nat Ecol Evol. 2023 Jun 15;7(8):1199–210. doi: 10.1038/s41559-023-02093-x (PMC10406605; doi:10.1038/s41559-023-02093-x)
Supplement: Supplementary file 1 — Supplementary Tables 1–4, Figs. 1–10 and Notes 1–6. [file 41559_2023_2093_MOESM1_ESM.pdf]

nature ecology & evolution

Article

https://doi.org/10.1038/s41559-023-02093-x

Biome-scale temperature sensitivity of ecosystem respiration revealed by atmospheric CO_2 observations

In the format provided by the authors and unedited

List of Supplementary Tables

1 Original estimates and optimal adjustments of the biome-scale temperature sensitivity of ecosystem respiration . . . 2
2 Correlations between domain-specific estimates of the temperature sensitivity of ecosystem respiration . . . 3
3 List of evaluated terrestrial biosphere models and data products . . . 4
4 List of sites where atmospheric CO_2 concentrations were measured . . . 7

List of Supplementary Figures

1 Optimal temperature sensitivities inferred from GPP combination tests . . . 11
2 Adjustments to model-specific activation energy in GPP combination tests . . . 12
3 Optimal temperature sensitivities inferred from the lateral flux test . . . 13
4 Adjustments to model-specific activation energy in the lateral flux test . . . 14
5 Model-represented temperature sensitivities with soil moisture sensitivity removed 15
6 Model-represented temperature sensitivities with radiation influences removed . 16
7 Model-represented temperature sensitivities with thermal acclimation accounted for 17
8 Multi-model mean seasonal cycles of GPP, ecosystem respiration, and NEE . . . 18
9 Multi-model mean seasonal cycles of GPP, rescaled ecosystem respiration, and the resulting NEE . . . 19
10 Comparison between climatological Q_{10} values at biome and plot scales . . . 20

List of Supplementary Notes

1 Combination tests to assess impact of the uncertainty in gross primary productivity on the inferred temperature sensitivities of ecosystem respiration . . . 21
2 Potential impact of lateral emissions on the inferred temperature sensitivities . . 21
3 Soil moisture influence on model-represented temperature sensitivities . . . 23
4 Radiation influence on model-represented temperature sensitivities . . . 23
5 Influences of acclimation on model-represented temperature sensitivities . . . 24
6 Analysis of the seasonal cycles of carbon fluxes . . . 25

Supplementary Table 1 | Original estimates of the temperature sensitivity of ecosystem respiration (E_a, eV) and optimal adjustments needed to match atmospheric CO_2 observations (ΔE_a, eV) for each model in North America and major biomes therein: croplands (CRO), evergreen needleleaf forests (ENF), and deciduous broadleaf and mixed forests (DBMF).

Ensemble	model	N. Am.		CRO		ENF		DBMF	
		E_a	ΔE_a	E_a	ΔE_a	E_a	ΔE_a	E_a	ΔE_a
MsTMIP v2	BIOME-BGC	0.33	0.06	0.24	0.01	0.31	0.11	0.28	0.10
	CLASS-CTEM-N	0.54	−0.10	0.41	−0.12	0.42	−0.04	0.43	−0.07
	CLM4	0.76	0.01	0.47	−0.05	0.33	0.05	0.37	0.07
	CLM4VIC	0.42	−0.01	0.49	−0.13	0.32	0.04	0.35	0.05
	DLEM	1.07	−0.16	0.78	−0.21	1.02	−0.24	0.92	−0.04
	GTEC	0.34	0.18	0.26	0.19	0.32	0.21	0.34	0.16
	ISAM	0.53	−0.29	0.64	−0.34	0.75	−0.29	0.67	−0.26
	JPL-HYLAND	1.19	−0.20	1.03	−0.25	0.84	−0.32	0.91	−0.18
	LPJ-wsl	0.65	−0.11	0.44	−0.13	0.79	−0.05	0.69	−0.09
	ORCHIDEE-LSCE	0.54	−0.09	0.53	−0.14	0.55	−0.04	0.50	−0.07
	SiB3	0.37	0.07	0.43	0.03	0.29	0.14	0.35	0.12
	SiBCASA	0.51	0.04	0.47	0.01	0.59	0.04	0.50	0.12
	TEM6	0.71	−0.07	0.49	−0.10	0.77	−0.06	0.66	−0.02
	TRIPLEX-GHG	1.04	−0.37	0.98	−0.43	1.06	−0.43	0.98	−0.35
	VEGAS	0.49	0.01	0.45	−0.04	0.46	0.04	0.47	0.03
	VISIT	0.44	−0.10	0.66	−0.24	0.47	−0.03	0.51	−0.06
TRENDY v6	CABLE	0.91	−0.30	0.73	−0.37	0.91	−0.21	0.79	−0.25
	CLASS-CTEM	0.43	−0.10	0.47	−0.18	0.38	−0.14	0.48	0.00
	CLM4.5	0.62	−0.01	0.62	−0.10	0.41	0.03	0.42	0.07
	DLEM	0.66	−0.07	0.76	−0.17	0.62	−0.03	0.64	0.02
	ISAM	0.88	−0.26	0.91	−0.33	1.02	−0.36	0.94	−0.19
	JULES	0.37	−0.05	0.54	−0.12	0.40	−0.01	0.41	−0.03
	LPJ-wsl	0.73	−0.17	0.72	−0.27	0.82	−0.03	0.76	−0.12
	OCN	0.53	−0.03	0.42	−0.05	0.47	0.00	0.46	−0.01
	ORCHIDEE	0.52	0.05	0.47	0.05	0.53	0.04	0.50	0.08
	ORCHIDEE-MICT	1.09	−0.12	0.66	−0.17	0.95	−0.11	0.75	−0.03
	SDGVM	0.54	0.02	0.60	−0.04	0.57	0.07	0.64	0.13
	VEGAS	0.45	0.02	0.43	−0.02	0.40	0.03	0.43	0.06
	VISIT	0.40	0.01	0.54	−0.08	0.47	0.09	0.43	0.06
FLUXCOM	ANN	0.48	−0.12	0.61	−0.19	0.53	−0.14	0.53	0.03
	MARS	0.54	−0.08	0.57	−0.15	0.54	−0.06	0.54	−0.01
	RF	0.61	−0.10	0.56	−0.14	0.63	−0.18	0.61	0.03

Supplementary Table 2 | Pearson's correlation coefficients (r) between original model estimates of the temperature sensitivity of ecosystem respiration (E_a, eV) in different domains. CRO, croplands; ENF, evergreen needleleaf forests; DBMF, deciduous broadleaf and mixed forests.

r	N. Am.	CRO	ENF
CRO	0.782		
ENF	0.847	0.765	
DBMF	0.860	0.857	0.967

Supplementary Table 3 | List of evaluated terrestrial biosphere models and data products.

Model	model full name	scenario[1]	references
**Multi-scale Synthesis and Terrestrial Model Intercomparison Project (MsTMIP) version 2**			
BIOME-BGC	n/a	BG1	Thornton et al. (2002)
CLASS-CTEM-N	Canadian Land Surface Scheme–Canadian Terrestrial Ecosystem Model (with Nitrogen dynamics)	BG1	Huang et al. (2011)
CLM4	Community Land Model version 4	BG1	Mao et al. (2012)
CLM4VIC	Community Land Model version 4 - Variable Infiltration Capacity	BG1	Lei et al. (2014)
DLEM	Dynamic Land Ecosystem Model	BG1	Tian et al. (2012)
GTEC	Global Terrestrial Ecosystem Carbon	SG3	Ricciuto et al. (2011)
ISAM	Integrated Science Assessment Model	BG1	Jain and Yang (2005) and El Masri et al. (2015)
JPL-HYLAND	n/a	SG3	Levy et al. (2004)
LPJ-wsl	Lund–Potsdam–Jena - Wald, Schnee, Landschaft	SG3	Sitch et al. (2003) and Bondeau et al. (2007)
ORCHIDEE-LSCE	Organising Carbon and Hydrology In Dynamic Ecosystems - Laboratoire des Sciences du Climat et de l'Environnement	SG3	Krinner et al. (2005)
SiB3	Simple Biosphere Model version 3	SG3	Baker et al. (2008) and Stöckli et al. (2008)
SiBCASA	Simple Biosphere Model - Carnegie–Ames–Stanford–Approach	SG3	Schaefer et al. (2008)
TEM6	Terrestrial Ecosystem Model version 6	BG1	McGuire et al. (2010)
TRIPLEX-GHG	n/a	BG1	Zhu et al. (2014)
VEGAS	Vegetation-Global-Atmosphere-Soil	SG3	Zeng et al. (2005), Zeng et al. (2014)
VISIT	Vegetation Integrative Simulator for Trace gases	SG3	Ito (2010)

continued on the next page

Model	model full name	scenario[1]	references
continued from the previous page			
**Trends in Net Land Atmosphere Exchange (TRENDY) version 6**			
CABLE	The Commonwealth Scientific and Industrial Research Organisation (CSIRO) Atmosphere Biosphere Land Exchange model	S3	Kowalczyk et al. (2006)
CLASS-CTEM	Canadian Land Surface Scheme–Canadian Terrestrial Ecosystem Model	S3	Arora (2003)
CLM4.5	Community Land Model version 4.5	S3	Oleson et al. (2013)
DLEM	Dynamic Land Ecosystem Model	S3	Tian et al. (2012)
ISAM	Integrated Science Assessment Model	S3	Jain and Yang (2005) and El Masri et al. (2015)
JULES	Joint UK Land Environment Simulator	S3	Best et al. (2011) and Clark et al. (2011)
LPJ-wsl	Lund–Potsdam–Jena - Wald, Schnee, Landschaft	S3	Sitch et al. (2003) and Bondeau et al. (2007)
OCN	Organising Carbon and Hydrology In Dynamic Ecosystems (with Carbon–Nitrogen coupling)	S3	Zaehle and Friend (2010)
ORCHIDEE	Organising Carbon and Hydrology In Dynamic Ecosystems	S3	Krinner et al. (2005)
ORCHIDEE-MICT	Organising Carbon and Hydrology In Dynamic Ecosystems - Ameliorated Interactions between Carbon and Temperature	S3	Guimberteau et al. (2018)
SDGVM	Sheffield Dynamic Global Vegetation Model	S3	Woodward et al. (1995)
VEGAS	Vegetation-Global-Atmosphere-Soil	S3	Zeng et al. (2005), Zeng et al. (2014)
VISIT	Vegetation Integrative Simulator for Trace gases	S3	Ito (2010)
continued on the next page			

Model	model full name	scenario[1]	references
continued from the previous page			
**FLUXCOM**			
ANN	Artificial Neural Networks	RS+METEO	Jung et al. (2020)
MARS	Multiple Adaptive Regression Splines	RS+METEO	Jung et al. (2020)
RF	Random Forests	RS+METEO	Jung et al. (2020)

[1]For the MsTMIP model ensemble, BG1 considers time-varying drivers of climate forcing, land-use history, atmospheric CO_2 concentrations, and nitrogen deposition, whereas SG3 is similar to BG1 but uses constant nitrogen deposition. For the TRENDY model ensemble, S3 considers time-varying climate forcing, atmospheric CO_2, and land-use history. For the FLUXCOM models, the RS+METEO setup uses both remote sensing and meteorological drivers for calculating fluxes.

Supplementary Table 4 | List of sites that provide atmospheric CO_2 concentration measurements in 2007–2010, compiled from the ObsPack GLOBALVIEWplus CO_2 (v3.2) data set.

ID	site name	latitude	longitude	start	end	height (m)	principal investigators
AAC	Austin Cary Memorial Forest, Gainesville, FL, USA	29.7381	−82.2188	2010	2010	32	Natasha Miles, Scott Richardson, and Ken Davis (PSU)
ACR	Chestnut Ridge, TX, USA	35.9311	−84.3324	2007	2010	61	
ACV	Canaan Valley, WV, USA	39.119	−79.4523	2007	2010	7	
AME	Mead, NE, USA	41.1649	−96.4701	2007	2010	4.5	
AOZ	Ozark, MO, USA	38.7441	−92.2	2007	2010	30	
FPK	Fort Peck, MT, USA	48.3079	−105.1017	2007	2008	3	
RCE	Centerville, IA, USA	40.7919	−92.8775	2007	2009	110	
RGV	Galesville, WI, USA	44.091	−91.3382	2007	2009	140	
RKW	Kewanee, IL, USA	41.2762	−89.9724	2007	2009	140	
RMM	Mead, NE, USA	41.1386	−96.4559	2007	2009	120	
RRL	Round Lake, MN, USA	43.5263	−95.4137	2007	2009	110	
AMT	Argyle, ME, USA	45.0346	−68.6821	2007	2010	107	Arlyn Andrews (NOAA/ESRL/GML)
BAO	Boulder Atmospheric Observatory, CO, USA	40.05	−105.004	2007	2010	300	
LEF	Park Falls, WI, USA	45.9453	−90.2744	2007	2010	396	
WBI	West Branch, IA, USA	41.7248	−91.3529	2007	2010	379	
WKT	Moody, TX, USA	31.3149	−97.3269	2007	2010	457	

continued on the next page

ID	site name	latitude	longitude	start	end	height (m)	principal investigators
continued from the previous page							
SNP	Shenandoah National Park, VA, USA	38.617	−78.35	2008	2010	17	Arlyn Andrews (NOAA/ESRL/GML) and Stephan De Wekker (UVA)
SCT	Beech Island, SC, USA	33.4057	−81.8334	2008	2010	305	Arlyn Andrews (NOAA/ESRL/GML) and Matt Parker (SRNL)
WGC	Walnut Grove, CA, USA	38.265	−121.4911	2007	2010	483	Arlyn Andrews (NOAA/ESRL/GML) and Marc Fischer (LBNL)
BRW	Barrow Atmospheric Baseline Observatory, AK, USA	71.323	−156.6114	2007	2010	16.5	Kirk Thoning and Pieter Tans (NOAA/ESRL/GML)
MVY	Marthas Vineyard, MA, USA	41.325	−70.5667	2007	2010	10	Colm Sweeney (NOAA/ESRL/GML)
BCK	Behchoko, NT, Canada	62.7979	−115.918	2010	2010	60	Doug Worthy (Environment Canada)
BRA	Bratt's Lake, SK, Canada	50.2016	−104.711	2009	2010	35	
CDL	Candle Lake, SK, Canada	53.9871	−105.1179	2007	2010	30	
CHM	Chibougamau, QC, Canada	49.6925	−74.3423	2007	2010	30	
EGB	Egbert, ON, Canada	44.231	−79.7838	2007	2010	3	
continued on the next page							

ID	site name	latitude	longitude	start	end	height (m)	principal investigators
continued from the previous page							
ESP	Estevan Point, BC, Canada	49.3829	−126.544	2009	2010	40	
EST	Esther, AB, Canada	51.67	−110.206	2010	2010	3	
ETL	East Trout Lake, SK, Canada	54.3537	−104.987	2007	2010	105	
FSD	Fraserdale, ON, Canada	49.8752	−81.5698	2007	2010	40	
LLB	Lac La Biche, AB, Canada	54.9538	−112.467	2007	2010	10	
WSA	Sable Island, NS, Canada	43.9323	−60.0126	2007	2010	25	
HFM	Harvard Forest, MA, USA	42.5378	−72.1714	2010	2010	29	Steve Wofsy and Bill Munger (Harvard)
SGP	Southern Great Plains, OK, USA	36.607	−97.489	2007	2010	60	Sebastien Biraud and Margaret Torn (LBNL)
HDP	Hidden Peak, UT, USA	40.56	−111.65	2007	2010	17.7	Britton Stephens (NCAR)
NWR	Niwot Ridge, CO, USA	40.0531	−105.5864	2007	2010	5.1	
RBA	Roof Butte, AZ	36.4614	−109.0956	2007	2010	21.9	
SPL	Storm Peak Laboratory, CO, USA	40.45	−106.73	2007	2010	9.1	
OFR	Fir, OR, USA	44.6465	−123.5514	2007	2010	38	Beverly Law (Oregon State U)
OMP	Mary's Peak, OR, USA	44.5043	−123.553	2007	2010	10	
OMT	Meolius, OR, USA	44.4524	−121.5572	2007	2010	33	
continued on the next page							

ID	site name	latitude	longitude	start	end	height (m)	principal investigators
continued from the previous page							
ONG	Burns, OR, USA	43.4704	−119.691	2007	2010	6	
OYQ	Yaquina Head, OR, USA	44.675	−124.067	2007	2010	12	
KCMP	Rosemount Research and Outreach Center, MN, USA	44.6886	−93.0728	2007	2008	200	Tim Griffis (UMN)

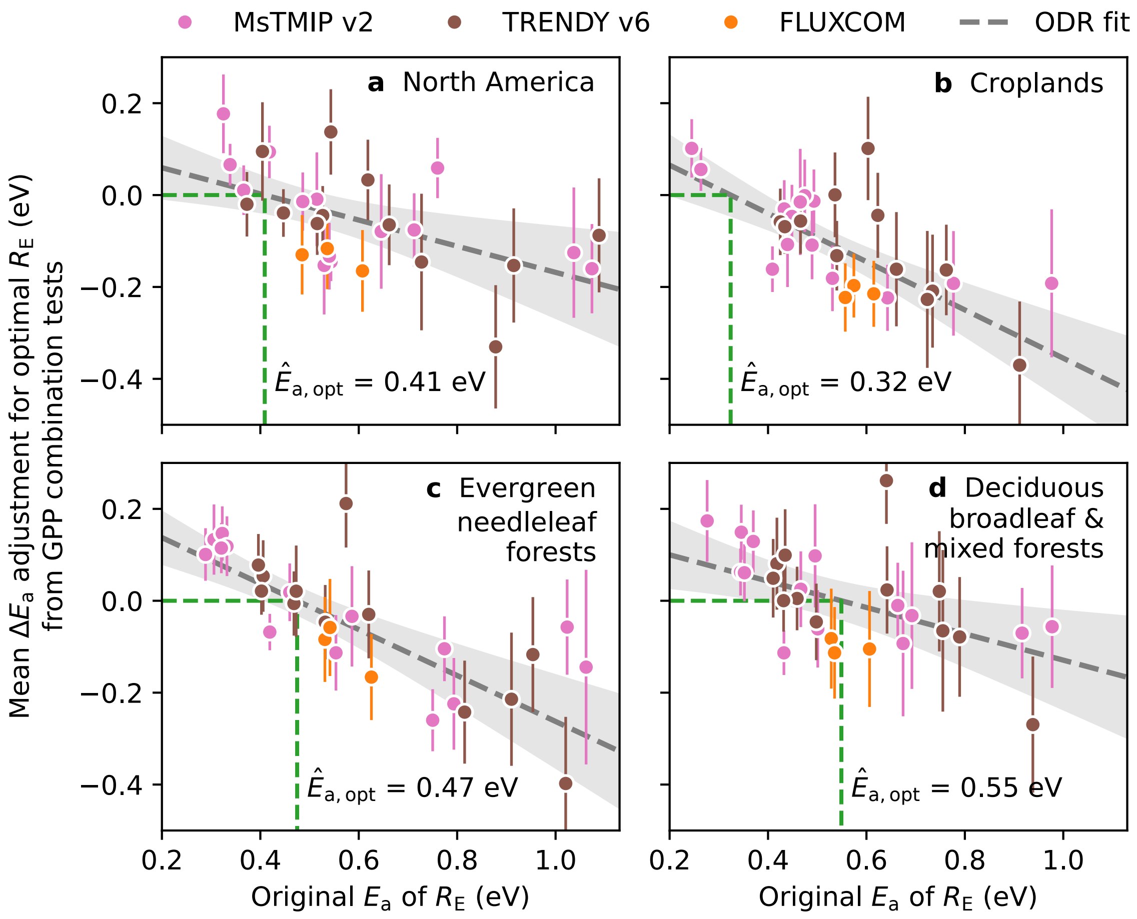

Supplementary Fig. 1 | Optimal North American and biome-specific temperature sensitivities of ecosystem respiration for individual models and the model ensemble based on GPP combination tests. Relationships between mean adjustments to model-specific estimates of the activation energy needed to maximize consistency with observed atmospheric CO_2 variability in GPP combination tests (ΔE_a, vertical axis) and the original estimates of activation energy (E_a, horizontal axis) for models in the MsTMIP v2 (pink), TRENDY v6 (brown), and FLUXCOM (orange) model ensembles in (**a**) the North American domain, (**b**) croplands, (**c**) evergreen needleleaf forests, and (**d**) deciduous broadleaf and mixed forests. Similar to Fig. 2 in the main text, only models for which the explanatory power of GPP estimates (R^2_{GPP}) exceeds that of shortwave radiation (R^2_{SW}) are included, yielding $N = 29$ models. For each model, the mean adjustment to the activation energy (vertical axis) is determined from averaging such adjustments from 28 "mix-and-match" pairs of GPP and ecosystem respiration estimates (see Supplementary Notes 1), with vertical error bars indicating ±1 standard deviation. The gray dashed lines represent the best ODR fit between ΔE_a and E_a estimates across the models, with light gray shading indicating the 95 % prediction interval. The optimal temperature sensitivity corresponds to the point where the ODR fit line crosses $\Delta E_a = 0$ eV (i.e., no adjustment to E_a is needed) and is indicated by a green dashed line. GPP, gross primary productivity; ODR, orthogonal distance regression.

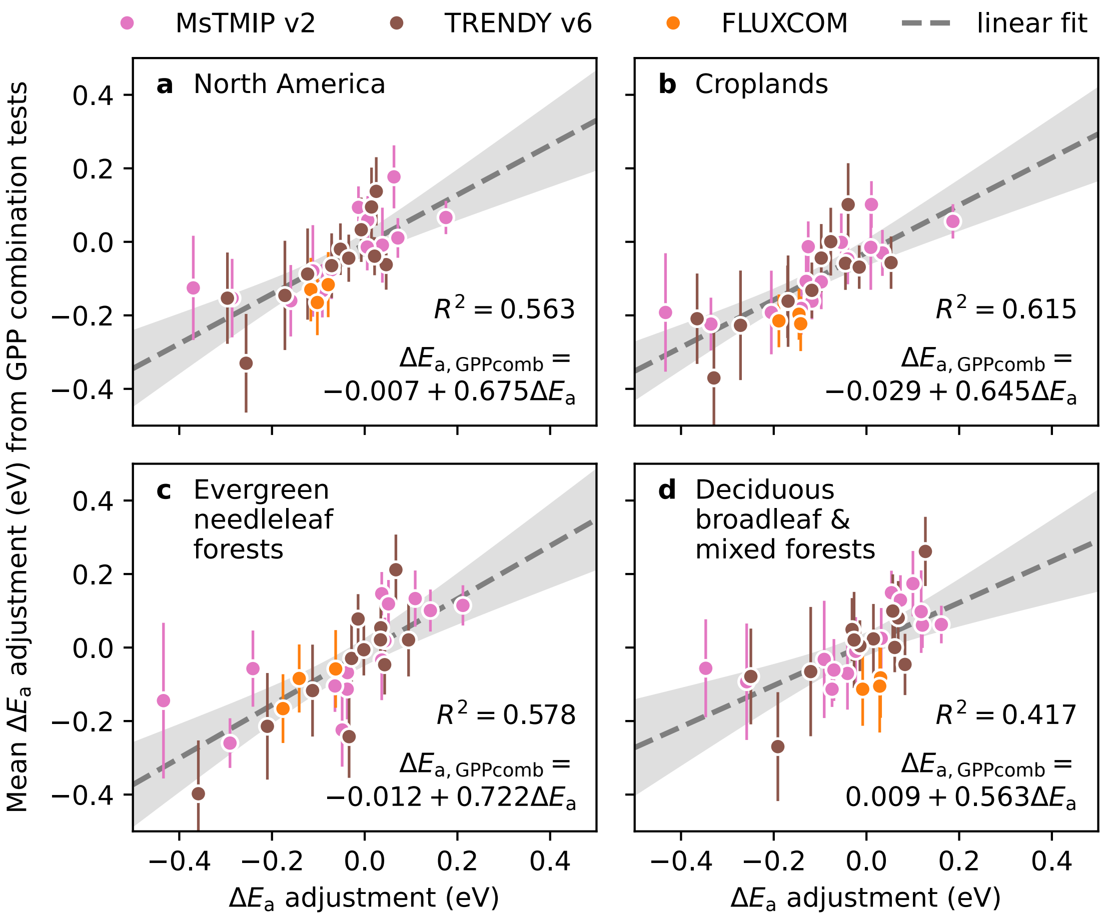

Supplementary Fig. 2 | Adjustments to model-specific estimates of activation energy in GPP combination tests agree well with those adjustments based on each model's original GPP estimates. Linear relationships between mean adjustments to model-specific estimates of the activation energy for ecosystem respiration needed to maximize consistency with observed atmospheric CO_2 variability in GPP combination tests (vertical axis) and those adjustments based on each model's original GPP estimates (horizontal axis) for models in the MsTMIP v2 (pink), TRENDY v6 (brown), and FLUXCOM (orange) model ensembles in (**a**) the North American domain, (**b**) croplands, (**c**) evergreen needleleaf forests, and (**d**) deciduous broadleaf and mixed forests. Same as Fig. 1, only models for which the explanatory power of GPP estimates exceeds that of shortwave radiation are included ($N = 29$). Vertical error bars indicating ±1 standard deviation among the adjustments derived from 28 "mix-and-match" pairs of GPP and ecosystem respiration estimates (see Supplementary Notes 1). Gray dashed lines are the best fit lines from ordinary least-squares linear regression, with light gray shading indicating the 95 % prediction interval. GPP, gross primary productivity.

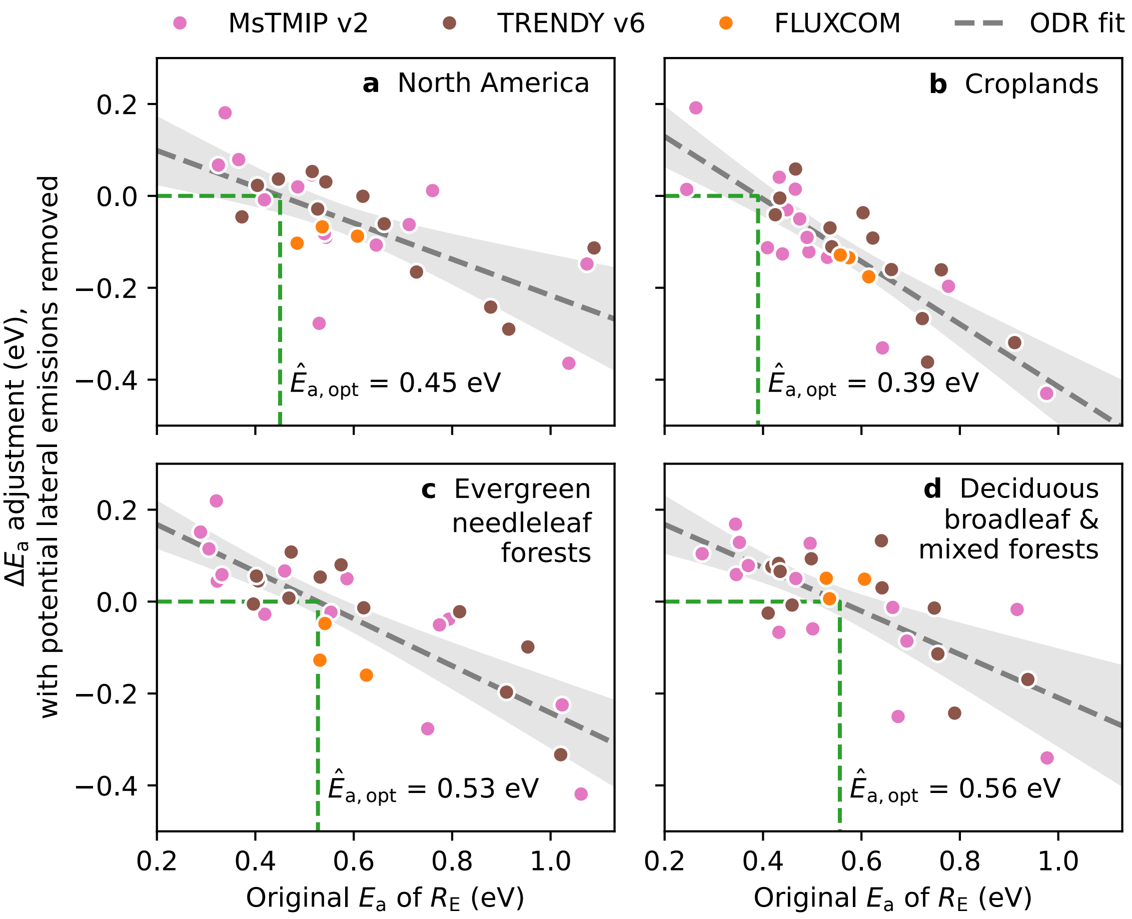

Supplementary Fig. 3 | Optimal North American and biome-specific temperature sensitivities of ecosystem respiration for individual models and the model ensemble based on a test of the potential impact of lateral fluxes. Relationships between adjustments to model-specific estimates of the activation energy needed to maximize consistency with observed atmospheric CO_2 variability in the presence of a synthetic field of potential lateral fluxes (ΔE_a, vertical axis; see Supplementary Notes 2) and the original estimates of activation energy (E_a, horizontal axis) for models in the MsTMIP v2 (pink), TRENDY v6 (brown), and FLUXCOM (orange) model ensembles in (**a**) the North American domain, (**b**) croplands, (**c**) evergreen needleleaf forests, and (**d**) deciduous broadleaf and mixed forests. Similar to Fig. 2 in the main text, only models for which the explanatory power of GPP estimates (R^2_{GPP}) exceeds that of shortwave radiation (R^2_{SW}) are included, yielding $N = 29$ models. The gray dashed lines represent the best ODR fit between ΔE_a and E_a estimates across the models, with light gray shading indicating the 95 % prediction interval. The optimal temperature sensitivity corresponds to the point where the ODR fit line crosses $\Delta E_a = 0$ eV (i.e., no adjustment to E_a is needed) and is indicated by a green dashed line. ODR, orthogonal distance regression.

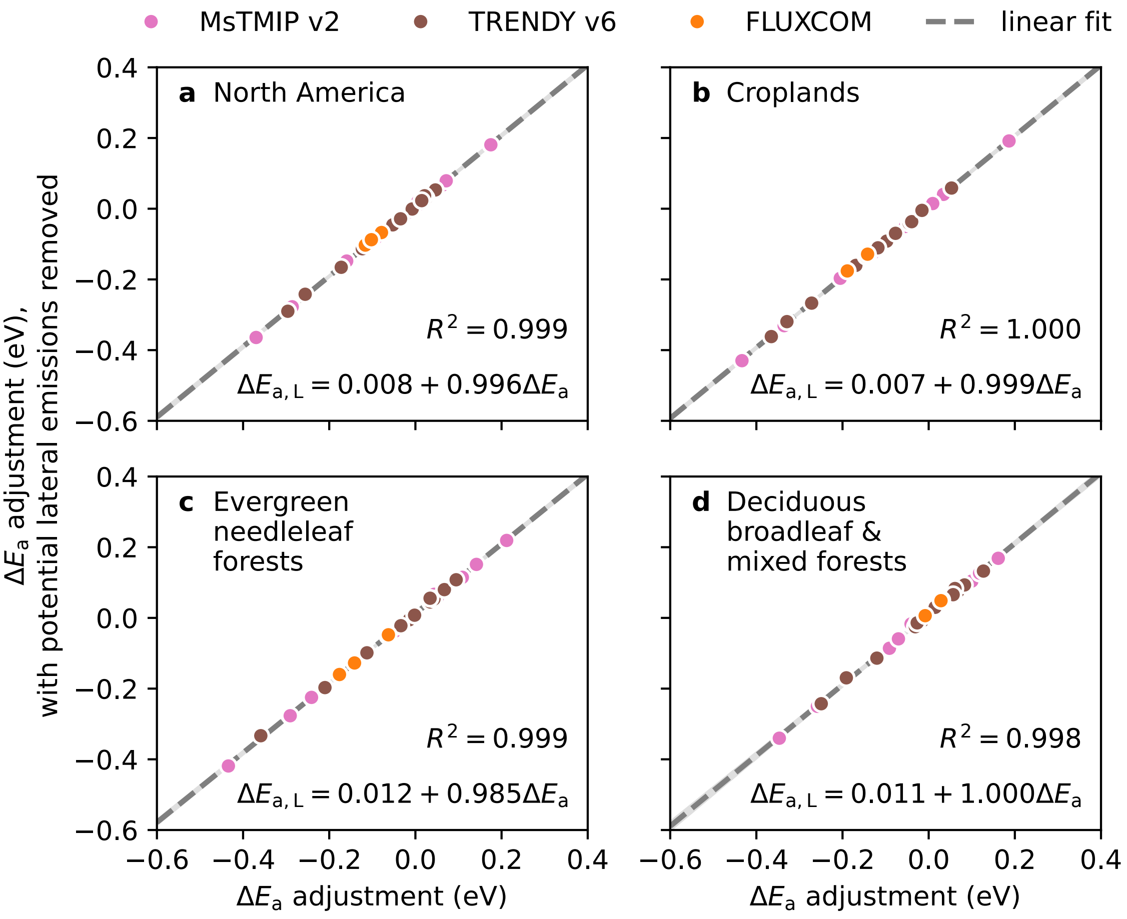

Supplementary Fig. 4 | Adjustments to model-specific estimates of activation energy in a test accounting for potential lateral fluxes agree well with the original adjustments that did not account for potential lateral fluxes. Linear relationships between adjustments to model-specific estimates of the activation energy for ecosystem respiration needed to maximize consistency with observed atmospheric CO_2 variability in the presence of a synthetic field of potential lateral fluxes (vertical axis; see Supplementary Notes 2) and those adjustments that did not account for potential lateral fluxes (horizontal axis) for models in the MsTMIP v2 (pink), TRENDY v6 (brown), and FLUXCOM (orange) model ensembles in (**a**) the North American domain, (**b**) croplands, (**c**) evergreen needleleaf forests, and (**d**) deciduous broadleaf and mixed forests. Same as Fig. 3, only models for which the explanatory power of GPP estimates exceeds that of shortwave radiation are included ($N = 29$). Gray dashed lines are the best linear fit lines from ordinary least-squares linear regression, with light gray shading indicating the 95 % prediction interval.

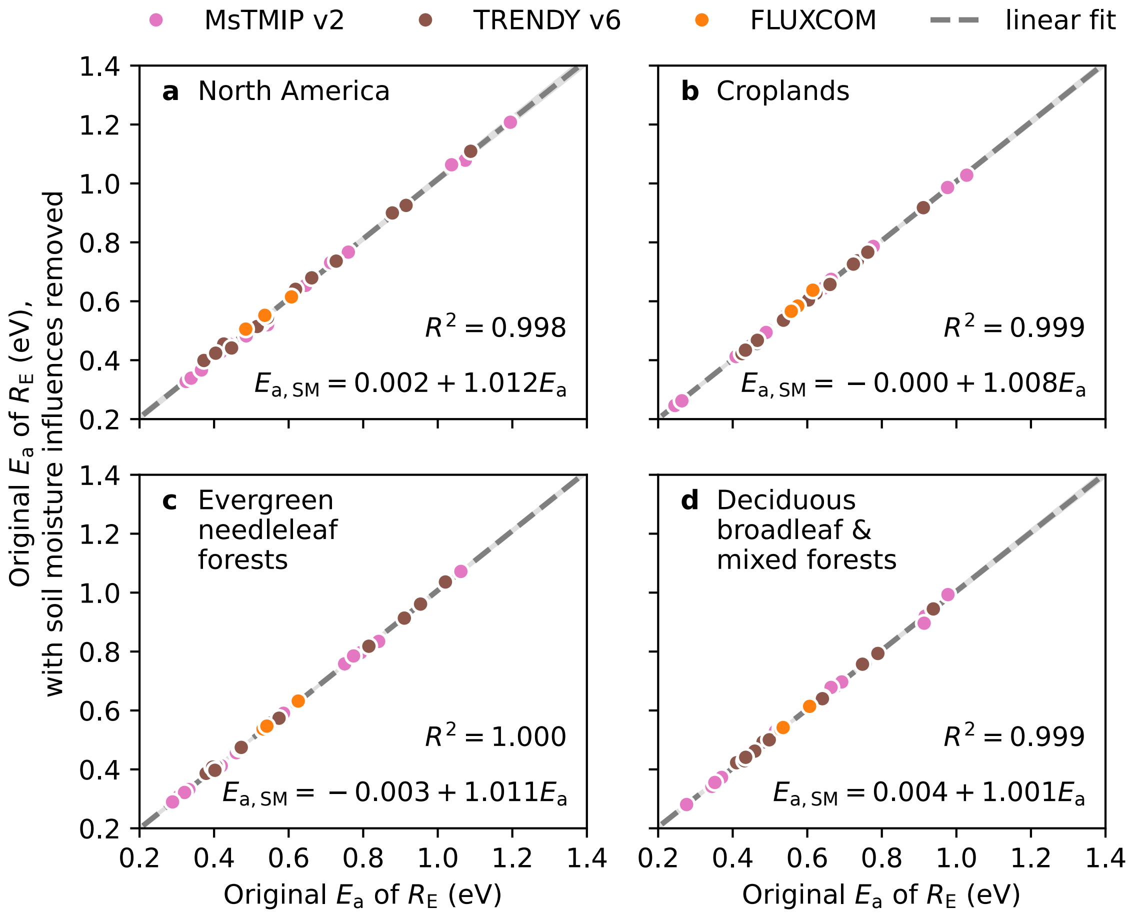

Supplementary Fig. 5 | Accounting for sensitivity to soil moisture explicitly does not yield different estimates of model-represented temperature sensitivities of ecosystem respiration. Model-represented temperature sensitivities of ecosystem respiration with soil moisture influences removed (vertical axis) agree with original estimates that do not separate soil moisture influences (horizontal axis) for models in the MsTMIP v2 (pink), TRENDY v6 (brown), and FLUXCOM (orange) model ensembles in (**a**) the North American domain, (**b**) croplands, (**c**) evergreen needleleaf forests, and (**d**) deciduous broadleaf and mixed forests. Gray dashed lines are the best linear fit lines from ordinary least-squares linear regression, with light gray shading indicating the 95 % prediction interval.

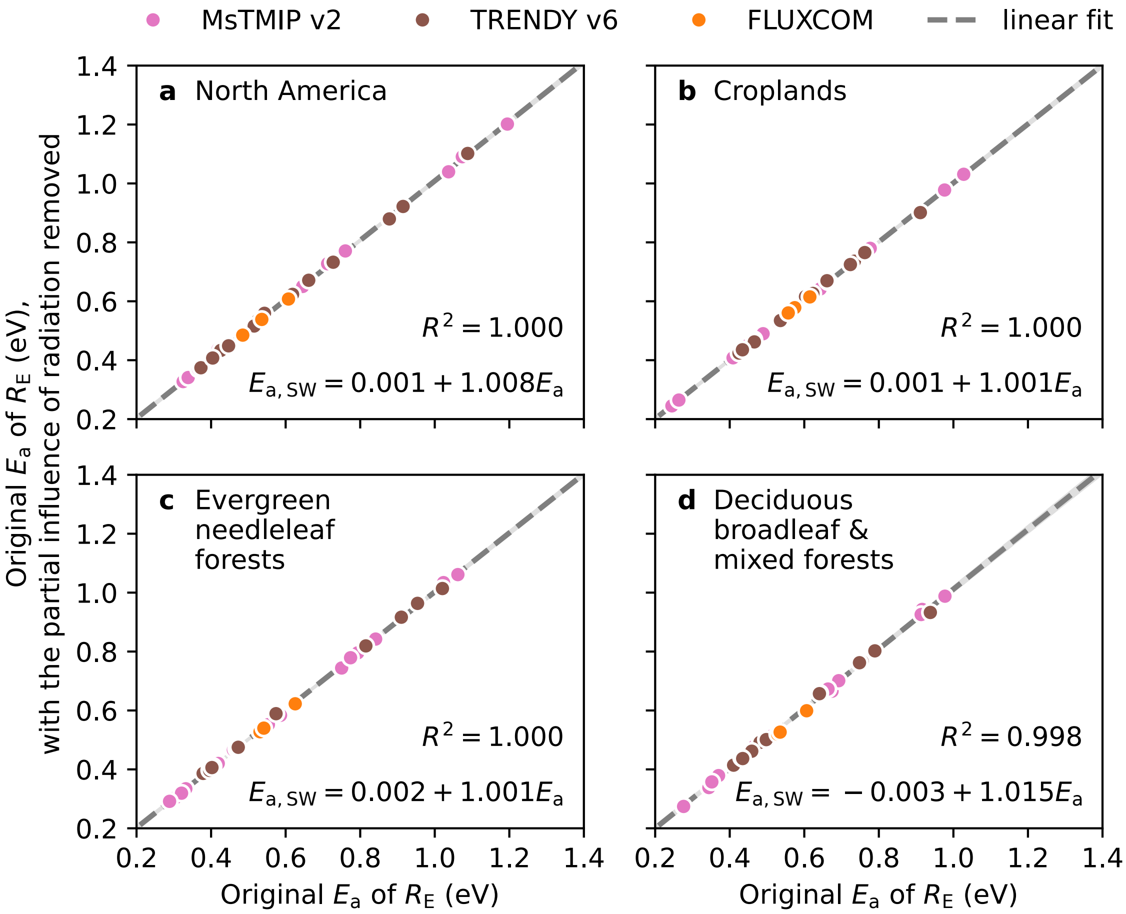

Supplementary Fig. 6 | Accounting for the partial influence of shortwave radiation does not impact estimates of model-represented temperature sensitivities of ecosystem respiration. Model-represented temperature sensitivities of ecosystem respiration with the partial influence of radiation removed (vertical axis) agree with original estimates that do not separate the influence of radiation (horizontal axis) for models in the MsTMIP v2 (pink), TRENDY v6 (brown), and FLUXCOM (orange) model ensembles in (**a**) the North American domain, (**b**) croplands, (**c**) evergreen needleleaf forests, and (**d**) deciduous broadleaf and mixed forests. Gray dashed lines are the best linear fit lines from ordinary least-squares linear regression, with light gray shading indicating the 95 % prediction interval.

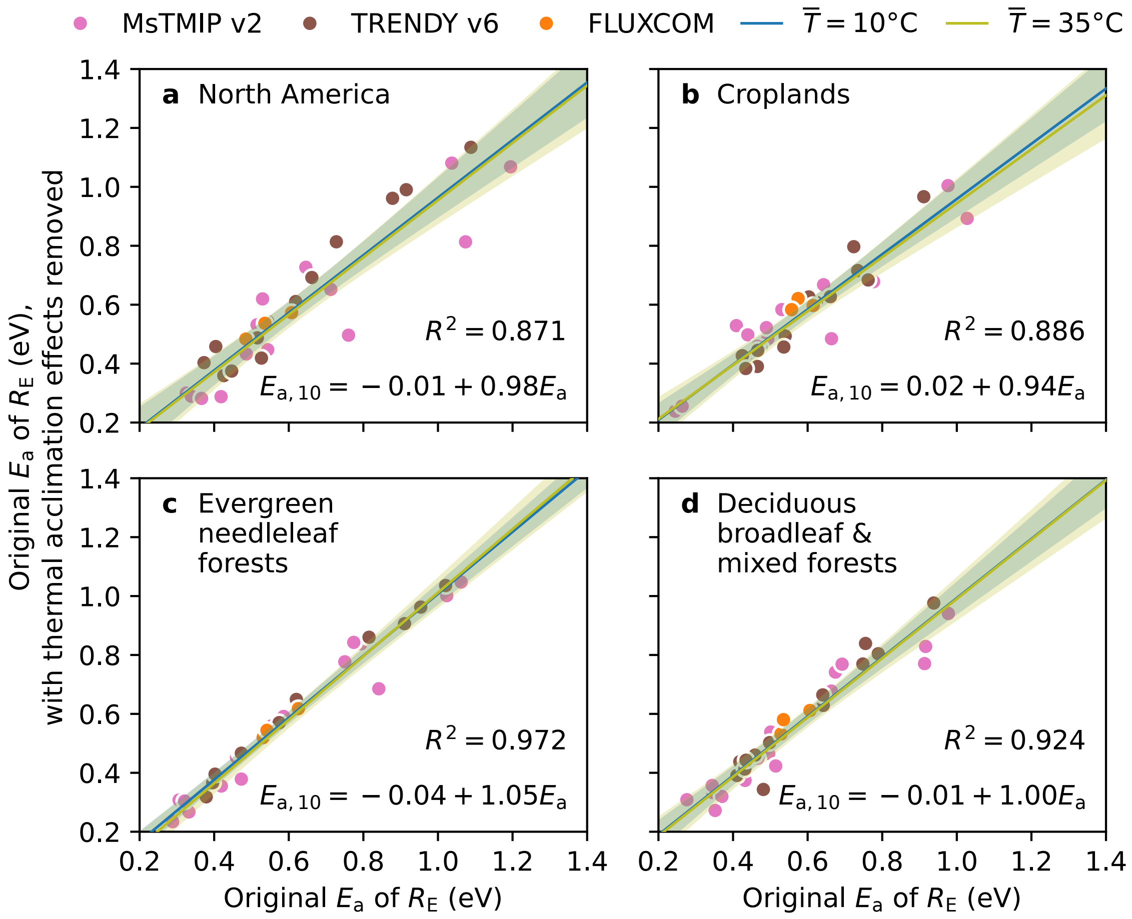

Supplementary Fig. 7 | Accounting for thermal acclimation effects does not yield significantly different estimates of model-represented temperature sensitivities of ecosystem respiration. Model-represented temperature sensitivities of ecosystem respiration with thermal acclimation effects accounted for (vertical axis) agree with original estimates that do not separately account for thermal acclimation effects (horizontal axis) for models in the MsTMIP v2 (pink), TRENDY v6 (brown), and FLUXCOM (orange) model ensembles in (**a**) the North American domain, (**b**) croplands, (**c**) evergreen needleleaf forests, and (**d**) deciduous broadleaf and mixed forests. Data points shown are temperature sensitivities ($E_{a,10}$) acclimated to a mean annual temperature of $\overline{T} = 10\,°C$ (see Supplementary Notes 5). Blue lines are the best fit lines, with light blue shade indicating the 95 % prediction intervals. Regression coefficients and coefficients of determination are shown for $\overline{T} = 10\,°C$. Due to the small thermal acclimation effect inferred from model-represented temperature sensitivities, data points of temperature sensitivities acclimated to different mean annual temperatures are not shown. Instead, similar regression lines for temperature sensitivities acclimated to a mean annual temperature of $\overline{T} = 35\,°C$ (an extreme case to illustrate the small differences) are shown in olive color for comparison, with 95 % prediction intervals.

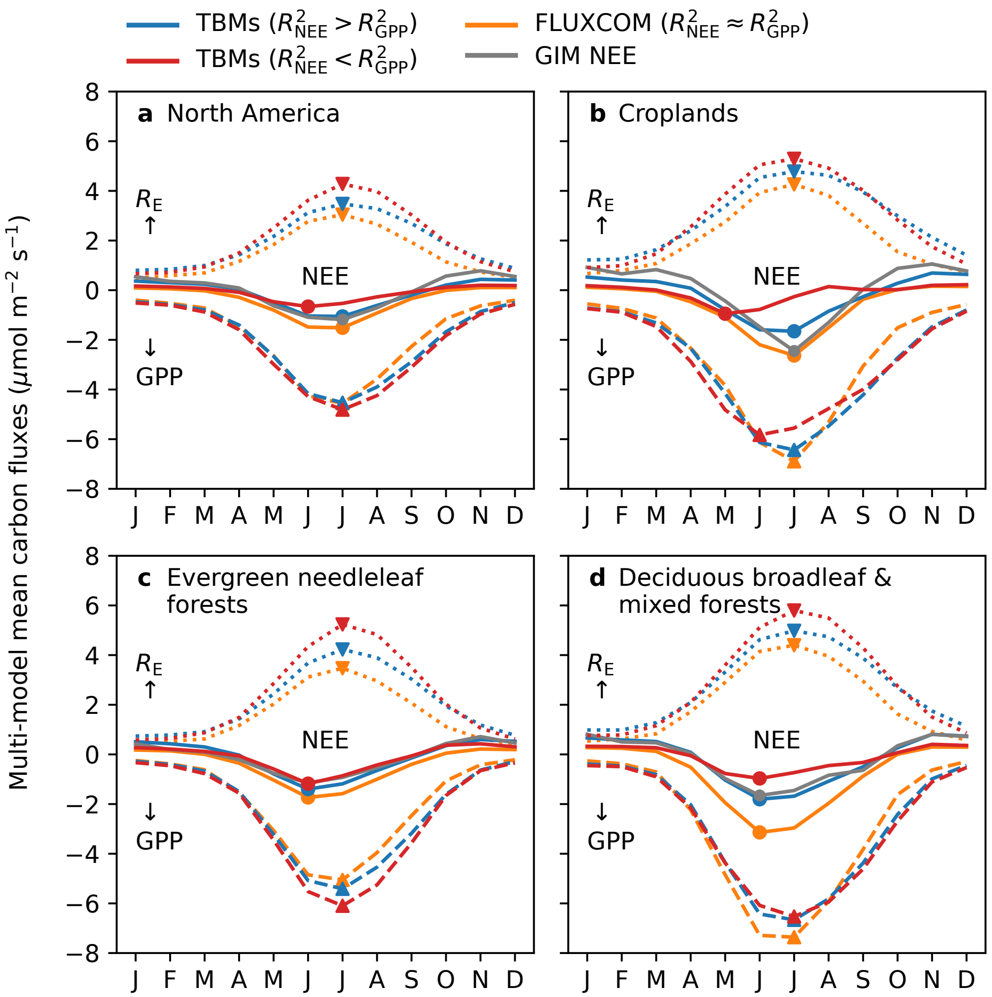

Supplementary Fig. 8 | Models for which NEE estimates explain atmospheric CO_2 variability less well than GPP estimates (red lines and symbols) show biased NEE seasonal cycles. Multi-model mean seasonal cycles of GPP (negative for uptake; dashed lines), ecosystem respiration (R_E; dotted lines), and NEE (negative for net uptake; solid lines) in (**a**) North America, (**b**) croplands, (**c**) evergreen needleleaf forests, and (**d**) deciduous broadleaf and mixed forests during 2007–2010 are shown for models for which the simulated GPP captures the variability in observed atmospheric CO_2 (R^2_{GPP}) better than does shortwave radiation ($R^2_{SW} = 0.23$). Blue lines indicate seasonal cycles of carbon fluxes from terrestrial biosphere models (TBMs) from the MsTMIP v2 and TRENDY v6 ensembles for which NEE estimates outperform GPP estimates in explaining the observed CO_2 variability ($R^2_{NEE} > R^2_{GPP}$; $N = 16$), whereas red lines represent seasonal cycles from TBMs for which GPP estimates outperform NEE estimates ($R^2_{NEE} < R^2_{GPP}$; $N = 10$). Seasonal cycles of carbon fluxes from the FLUXCOM models ($N = 3$) are in orange. In addition, the geostatistical inverse model estimates of NEE (GIM NEE) are shown as a reference (gray). Months of peak fluxes are indicated by circles (NEE), upward triangles (GPP), and downward triangles (R_E). GPP, gross primary productivity; NEE, net ecosystem exchange.

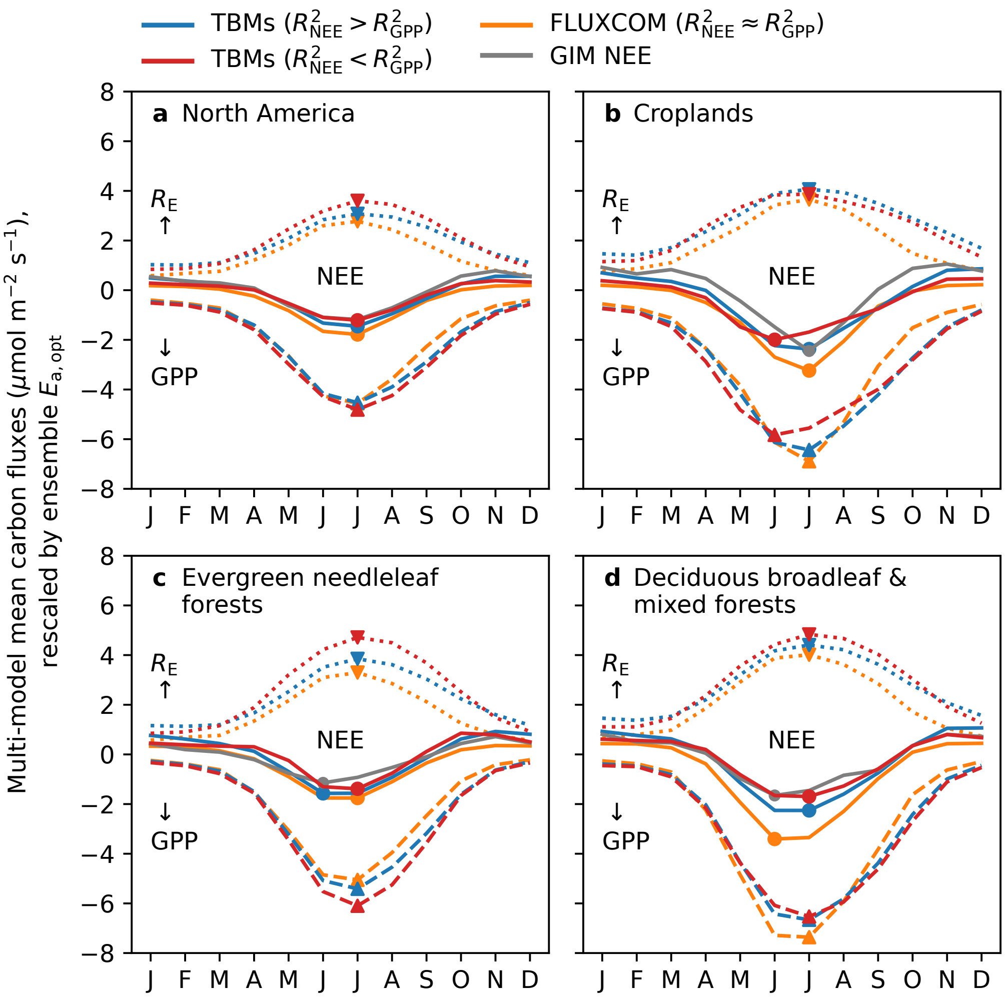

Supplementary Fig. 9 | Models for which NEE estimates explain atmospheric CO_2 variability less well than GPP estimates (red lines and symbols) show improved NEE seasonal cycles after rescaling ecosystem respiration estimates using the optimal temperature sensitivity for North America. Multi-model mean seasonal cycles of GPP (dashed lines), ecosystem respiration rescaled using the optimal temperature sensitivity for North America (R_E; dotted lines), and the resultant NEE (solid lines) in (**a**) North America, (**b**) croplands, (**c**) evergreen needleleaf forests, and (**d**) deciduous broadleaf and mixed forests during 2007–2010. Similar to Fig. 8, only models for which GPP estimates explain observed atmospheric CO_2 variability better than shortwave radiation ($R^2_{GPP} > R^2_{SW} = 0.23$) are included. Blue lines indicate seasonal cycles of carbon fluxes from TBMs for which the explanatory power of original NEE estimates exceeds that of GPP estimates ($R^2_{NEE} > R^2_{GPP}$; $N = 16$), whereas red lines represent seasonal cycles from TBMs for which NEE originally trails GPP in explanatory power ($R^2_{NEE} < R^2_{GPP}$; $N = 10$). Seasonal cycles of carbon fluxes from the FLUXCOM models ($N = 3$) are in orange. In addition, GIM NEE is shown as a reference (gray). Months of peak fluxes are indicated by circles (NEE), upward triangles (GPP), and downward triangles (R_E). GPP, gross primary productivity; NEE, net ecosystem exchange.

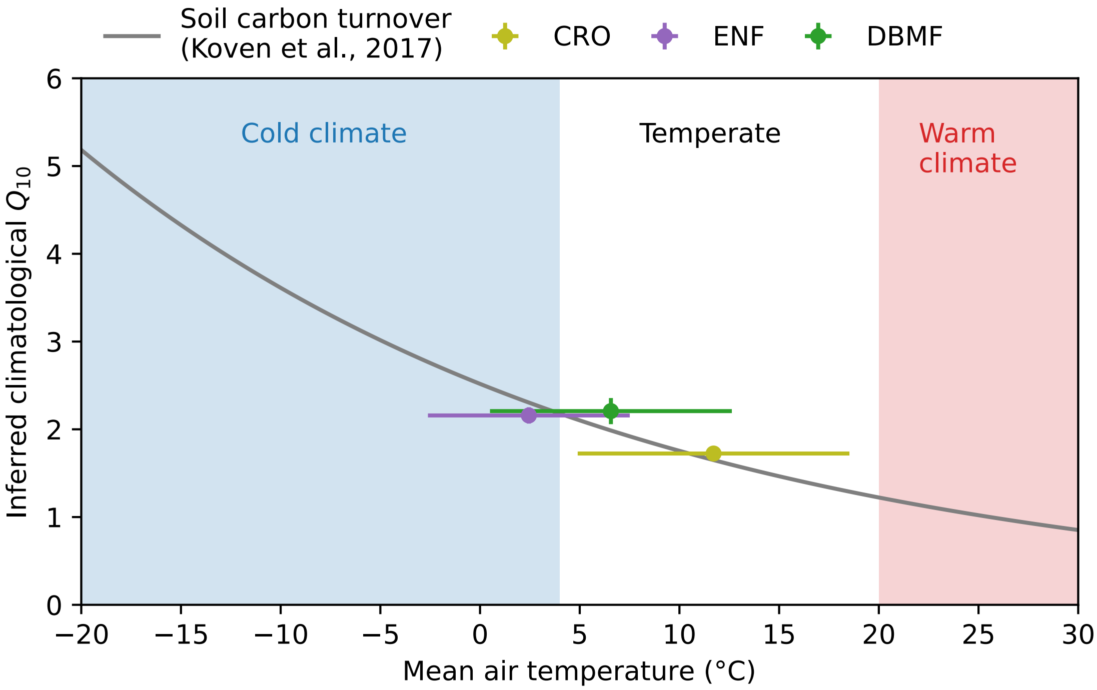

Supplementary Fig. 10 | Inferred Q_{10} values for biome-scale ecosystem respiration are consistent with the relationship between climatological Q_{10} values for heterotrophic respiration derived from field observations and mean annual air temperature. Optimal temperature sensitivities of ecosystem respiration for croplands (CRO; olive color), evergreen needleleaf forests (ENF; purple), and deciduous broadleaf and mixed forests (DBMF; green) are converted to equivalent Q_{10} values following Eq. (3) in the main text and plotted against mean annual air temperature within these biomes. Vertical error bars indicate ±1 standard error of Q_{10} estimates (based on $N = 29$ independent estimates in Fig. 2), whereas horizontal error bars indicate ±1 standard deviation of mean annual air temperature (based on 328 grid cells for croplands, 544 for evergreen needleleaf forests, and 357 for deciduous broadleaf and mixed forests). Also shown is the relationship between climatological Q_{10} for heterotrophic respiration derived from field observations and mean annual air temperature (gray line) from Koven et al. (2017) for comparison. Blue and red shaded areas indicate high-sensitivity, cold-climate emergent domain and low-sensitivity, warm-climate emergent domain (Koven et al., 2017), respectively.

Notes 1 Combination tests to assess impact of the uncertainty in gross primary productivity on the inferred temperature sensitivities of ecosystem respiration

To test the influence of uncertainty in model estimates of gross primary productivity (GPP) on the inferred temperature sensitivities of ecosystem respiration, we mix and match GPP and ecosystem respiration estimates drawn from different model simulations. For each "mix-and-match" pair we recalculate the adjustment to the activation energy of ecosystem respiration in the same way as described in the Methods in the main text (see "Optimizing the temperature sensitivity of respiration"). For quality control, the combination tests are limited to the 29 model simulations for which the explanatory power of GPP estimates (R^2_{GPP}) exceeds that of shortwave radiation (R^2_{SW}; Extended Data Fig. 6), consistent with Fig. 2 in the main text. This means that for each model simulation, the estimates of ecosystem respiration are paired with GPP estimates from each of the other 28 model simulations to obtain a set of optimal adjustments to the activation energy of ecosystem respiration under different GPP scenarios. Then, we obtain the average adjustment for each model simulation. Lastly, the North American and biome-specific optimal temperature sensitivities are derived from the same orthogonal distance regression method used to obtain the main results (Fig. 2 in the main text).

Results from the combination tests confirm that GPP uncertainty does not substantially impact the inferred optimal temperature sensitivities of ecosystem respiration. Differences between the optimal temperature sensitivities derived from the combination tests (Supplementary Fig. 1) and those derived based on models' original GPP estimates (Fig. 2, main text) are well within the uncertainty range. The optimal temperature sensitivity for croplands derived from the combination tests (0.32 eV; Supplementary Fig. 1b) shows the largest difference from the original optimal estimate (0.38 eV). However, this is not surprising given that croplands are the largest contributor to model divergence in North American GPP estimates (Sun et al., 2021). Adjustments to estimates of activation energy for individual models in the combination tests are also highly consistent with adjustments based on each model's original GPP estimates (Supplementary Fig. 2).

Notes 2 Potential impact of lateral emissions on the inferred temperature sensitivities

Terrestrial biosphere models (TBMs) vary in terms of their representation of carbon emissions from lateral transport processes (Huntzinger et al., 2013). Chief among these processes are consumption of crop products, decay of wood products, and outgassing from inland waters (Ciais et al., 2021). It remains difficult to incorporate these flux components into the evaluation of carbon budgets due to the lack of well-validated gridded observational data sets. Here, we construct a synthetic field of plausible emissions from lateral fluxes and evaluate the impact of these emissions on the

inferred temperature sensitivities of respiration for North America and individual biomes. Because TBMs may resolve different components of lateral emissions and it is challenging to harmonize model differences, this test is intended to give an upper bound of the potential impact of lateral emissions.

We construct the synthetic lateral emission field based on the magnitudes of North American crop product (182 $Tg\,C\,yr^{-1}$), wood product (76 $Tg\,C\,yr^{-1}$), and riverine (219 $Tg\,C\,yr^{-1}$) emissions compiled in Ciais et al. (2021) and the spatial distribution of crop, wood, and riverine lateral fluxes in Byrne et al. (2023). First, we assume that the lateral fluxes that represent additional efflux of carbon, i.e., carbon transported into a region from elsewhere (for example, crop and wood products shipped to population centers), are fully transformed into CO_2 emissions. Following this assumption, we use the spatial distribution of lateral fluxes from crop trade, wood trade, and riverine transport (Byrne et al., 2022; Byrne et al., 2023) to represent the spatial distribution of corresponding emissions. We then scale these emissions to the magnitudes given in Ciais et al. (2021). Due to the lack of monthly resolved emissions, we distribute lateral emissions equally across months. The resulting lateral emission field is then converted to atmospheric CO_2 enhancements using the same atmospheric transport footprints used for the main results (see "Optimizing the temperature sensitivity of respiration" in the Methods). We then subtract the influence of lateral emissions (mean: 0.27 ppm; median: 0.20 ppm) from atmospheric CO_2 observations. Finally, we perform the same optimization procedure to infer the optimal temperature sensitivities as we did for the main results (Fig. 2).

Inferred optimal temperature sensitivities for North America and individual biomes are indistinguishable from those derived without considering potential lateral emissions (Supplementary Fig. 3). The largest differences in the optimal temperature sensitivity estimate are found in forests (0.03 eV) but are still within the uncertainty range. Unsurprisingly, adjustments to estimates of activation energy for individual models in the lateral emission test also agree with adjustments without considering potential lateral emissions (Supplementary Fig. 4).

These results suggest that different ways of accounting lateral emission (or lack thereof) in TBMs are unlikely to meaningfully affect inferred temperature sensitivities of ecosystem respiration at the biome scale.

Notes 3 Soil moisture influence on model-represented temperature sensitivities

To account for the potential influence of soil moisture on the temperature sensitivity of ecosystem respiration, we fit the following regression:

$$\ln R_\mathrm{E} = \beta_0 + \beta_1 \cdot \frac{1}{T} + \beta_2 \cdot \theta + \epsilon, \tag{1}$$

where R_E (µmol m^{-2} s^{-1}) is model-simulated ecosystem respiration, θ (m^3 m^{-3}) is the mean volumetric soil water content in the top 2 m, obtained from North American Regional Reanalysis (Mesinger et al., 2006), β_0, β_1, and β_2 are the coefficients, and ϵ is the residual. From the coefficient of $1/T$ we obtain the temperature sensitivity of ecosystem respiration, $E_{\mathrm{a,SM}}$ (eV), with soil moisture influences removed.

We find that accounting for soil moisture influences does not yield different estimates of model-represented temperature sensitivities of ecosystem respiration (Supplementary Fig. 5). Although soil moisture modifies the temperature sensitivity of ecosystem respiration at the plot scale and on weekly to seasonal timescales (Reichstein et al., 2002), this effect is minor at the biome scale and over a longer time horizon. Thus, misidentification of the temperature sensitivity due to misrepresented soil moisture sensitivity seems unlikely.

Notes 4 Radiation influence on model-represented temperature sensitivities

Because shortwave radiation is highly correlated with temperature at a monthly resolution and on the biome scale, we take a two-step regression approach to account for the partial influence of shortwave radiation on model-represented temperature sensitivities of ecosystem respiration. To remove the co-variability between shortwave raidation and temperature, we first fit the following regression:

$$R_\mathrm{sw} = \alpha_0 + \alpha_1 \cdot \frac{1}{T} + \epsilon_{R_\mathrm{sw}}, \tag{2}$$

where R_sw is the downward shortwave radiation flux at the surface (W m^{-2}) obtained from North American Regional Reanalysis (Mesinger et al., 2006), α_0 and α_1 are regression coefficients, and ϵ_{R_sw} is the residual.

We then fit the following regression to account for the partial influence of shortwave radiation in determining the temperature sensitivity:

$$\ln R_\mathrm{E} = \beta_0 + \beta_1 \cdot \frac{1}{T} + \beta_2 \cdot \epsilon_{R_\mathrm{sw}} + \epsilon, \tag{3}$$

where β_0, β_1, and β_2 are regression coefficients and ϵ is the residual. Similarly, from the coefficient

of $1/T$ we obtain the temperature sensitivity of ecosystem respiration, $E_{a,SW}$ (eV), with the partial influence of shortwave radiation removed.

We find that removing the partial influence of shortwave radiation does not meaningfully alter model-represented temperature sensitivities of ecosystem respiration on the biome scale (Supplementary Fig. 6). Although radiation is often examined as a covariate of ecosystem respiration (Jung et al., 2017), its causal link to ecosystem respiration is mediated by its effects on GPP (Monteith, 1972) and the correlation between GPP and ecosystem respiration (Baldocchi, 2008). Consequently, the absence of a substantial influence of GPP bias on the inferred temperature sensitivity of biome-scale ecosystem respiration (Supplementary Fig. 1) indicates that it is unlikely that radiation would bias the inferred temperature sensitivities.

Notes 5 Influences of acclimation on model-represented temperature sensitivities

Plants and microbes have been found to physiologically adjust the temperature response of respiration according to ambient temperature, known as thermal acclimation (Atkin & Tjoelker, 2003; Bradford et al., 2008). To account for the impact of potential thermal acclimation on the temperature sensitivity of ecosystem respiration, we assume that the activation energy (E_a, eV) is a linear function of the mean annual temperature:

$$E_a\left(\overline{T}\right) = E_{a,0} + \Delta C_p\left(\overline{T} - \overline{T}_0\right), \tag{4}$$

where ΔC_p (eV K^{-1}) is the linear temperature response of E_a, $\overline{T}$ (K) is the mean annual temperature, $E_{a,0}$ (eV) is the temperature sensitivity at a reference mean annual temperature $\overline{T}_0 = 273.15$ K. This also means that

$$\frac{\partial E_a}{\partial \overline{T}} = \Delta C_p. \tag{5}$$

Substituting Eq. 4 in the Arrhenius equation for ecosystem respiration (Eq. 1 in the main text), we obtain

$$\ln R_E = \ln A - \frac{E_a + \Delta C_p(\overline{T} - \overline{T}_0)}{k_B T} = \ln A - \frac{E_a}{k_B}\left(\frac{1}{T}\right) - \frac{\Delta C_p}{k_B}\left(\frac{\overline{T} - \overline{T}_0}{T}\right) \tag{6}$$

where A is the pre-exponential factor in the Arrhenius equation.

Let

$$\begin{cases} x_1 = \dfrac{1}{T}, \\ x_2 = \dfrac{\overline{T} - \overline{T}_0}{T}. \end{cases} \tag{7}$$

We then fit the regression

$$\ln R_\mathrm{E} \sim x_1 + x_2. \tag{8}$$

From the coefficient of x_1 we obtain the temperature sensitivity of ecosystem respiration at the reference mean annual temperature, $E_{\mathrm{a},0}$. From the coefficient of x_2 we obtain ΔC_p, the temperature response of E_a.

Accounting for the thermal acclimation effect does not yield substantially different estimates of model-represented temperature sensitivities (Supplementary Fig. 7). Therefore, thermal acclimation of respiration does not explain the spread in model-represented temperature sensitivity of ecosystem respiration, or the differences in such sensitivity between plot and biome scales and across biomes.

Notes 6 Analysis of the seasonal cycles of carbon fluxes

We find strong differences, both in terms of seasonality and magnitude, in estimates of net ecosystem exchange (NEE), gross primary productivity (GPP), and ecosystem respiration (R_E) between the group of TBMs for which NEE estimates explain atmospheric CO_2 variability better than those models' GPP estimates ($R^2_\mathrm{NEE} > R^2_\mathrm{GPP}$) and the group of TBMs for which moving from GPP to NEE degrades performance ($R^2_\mathrm{NEE} < R^2_\mathrm{GPP}$). The differences in the seasonal cycle of NEE between these two groups are especially notable in the croplands (Fig. 8b), showing up as a two-month early bias of the peak timing of NEE for TBMs for which GPP outperforms NEE ($R^2_\mathrm{NEE} < R^2_\mathrm{GPP}$; red lines and symbols) relative to that of the geostatistical inverse model estimates of NEE (GIM NEE; gray line and symbols; Shiga et al., 2018). This phase bias in cropland NEE is caused by both an offset of the timing of peak GPP and an overestimate of the seasonal amplitude of ecosystem respiration relative to TBMs for which NEE explains observed atmospheric variability better than GPP ($R^2_\mathrm{NEE} > R^2_\mathrm{GPP}$; blue lines and symbols) and FLUXCOM models (orange lines and symbols; Fig. 8b). By contrast, the seasonal cycles of NEE in deciduous broadleaf and mixed forests (Fig. 8d) do not show a phase bias but differ strongly in amplitude across model groups. Model estimates of NEE in evergreen needleleaf forests do not differ much among different model groups (Fig. 8c).

Analysis of the seasonal cycles of carbon fluxes further implicates the seasonal amplitude of ecosystem respiration estimates (Fig. 8; dotted lines) as a source of the NEE phase bias that degrades performance. For TBMs for which GPP outperforms NEE in explaining atmospheric CO_2 variability ($R^2_\mathrm{NEE} < R^2_\mathrm{GPP}$), the one-month early bias in the peak timing of cropland GPP relative to the rest of TBMs and FLUXCOM models (Fig. 8b; red dashed lines) is amplified to a two-month bias in the peaking timing of NEE (red solid lines) by the high bias in the seasonal amplitude of

ecosystem respiration (red dotted lines). This is also true for the North American domain (Fig. 8a), where the high bias in the seasonal amplitude of ecosystem respiration causes a phase bias in NEE for TBMs for which GPP outperforms NEE ($R^2_{NEE} < R^2_{GPP}$), despite the fact that the seasonal cycles of GPP are nearly identical between the two groups of models. In forested biomes (Fig. 8c, d), there is also a consistent high bias in the seasonal amplitude of ecosystem respiration for TBMs for which GPP outperforms NEE ($R^2_{NEE} < R^2_{GPP}$), even though this bias does not cause a phase bias in NEE. Moreover, for these TBMs ($R^2_{NEE} < R^2_{GPP}$), the high bias in peak respiration is always accompanied by a smaller but noticeable low bias in winter respiration relative to TBMs for which NEE outperforms GPP ($R^2_{NEE} > R^2_{GPP}$) and relative to FLUXCOM models (Fig. 8). This suggests that the amplitude bias in ecosystem respiration stems from the temperature sensitivities of ecosystem respiration as represented within each model.

Rescaling ecosystem respiration by the ensemble-optimal temperature sensitivity over North America (0.43 eV) leads to reduced bias in both the seasonal amplitude of ecosystem respiration and the timing of peak NEE for models for which NEE performance trailed that of GPP ($R^2_{NEE} < R^2_{GPP}$; red lines and symbols) in the North American domain (Fig. 9a) and croplands (Fig. 9b). For these models, the NEE phase bias relative to GIM NEE is eliminated over North America and reduced to one month in croplands after rescaling. In forested biomes (Fig. 9c, d), however, rescaling causes inconsistent shifts of the NEE peak from June to July. But this is mainly due to the small difference in NEE between June and July in forested biomes. Overall, the comparison of the seasonal cycles of NEE and ecosystem respiration before and after the rescaling lends strong support to the finding that bias in the temperature sensitivity of ecosystem respiration is a leading cause of NEE underperformance.

References

Arora, V. K. (2003). Simulating energy and carbon fluxes over winter wheat using coupled land surface and terrestrial ecosystem models. *Agricultural and Forest Meteorology*, *118*(1-2), 21–47. https://doi.org/10.1016/s0168-1923(03)00073-x

Atkin, O. K., & Tjoelker, M. G. (2003). Thermal acclimation and the dynamic response of plant respiration to temperature. *Trends in Plant Science*, *8*(7), 343–351. https://doi.org/10.1016/S1360-1385(03)00136-5

Baker, I. T., Prihodko, L., Denning, A. S., Goulden, M., Miller, S., & da Rocha, H. R. (2008). Seasonal drought stress in the Amazon: Reconciling models and observations. *Journal of Geophysical Research: Biogeosciences*, *113*(G1), G00B01. https://doi.org/10.1029/2007jg000644

Baldocchi, D. (2008). “Breathing” of the terrestrial biosphere: Lessons learned from a global network of carbon dioxide flux measurement systems. *Australian Journal of Botany*, *56*(1), 1–26. https://doi.org/10.1071/bt07151

Best, M. J., Pryor, M., Clark, D. B., Rooney, G. G., Essery, R. L. H., Ménard, C. B., Edwards, J. M., Hendry, M. A., Porson, A., Gedney, N., Mercado, L. M., Sitch, S., Blyth, E., Boucher, O., Cox, P. M., Grimmond, C. S. B., & Harding, R. J. (2011). The Joint UK Land Environment Simulator (JULES), model description – part 1: Energy and water fluxes. *Geoscientific Model Development*, *4*(3), 677–699. https://doi.org/10.5194/gmd-4-677-2011

Bondeau, A., Smith, P. C., Zaehle, S., Schaphoff, S., Lucht, W., Cramer, W., Gerten, D., Lotze-Campen, H., Müller, C., Reichstein, M., & Smith, B. (2007). Modelling the role of agriculture for the 20th century global terrestrial carbon balance. *Global Change Biology*, *13*(3), 679–706. https://doi.org/10.1111/j.1365-2486.2006.01305.x

Bradford, M. A., Davies, C. A., Frey, S. D., Maddox, T. R., Melillo, J. M., Mohan, J. E., Reynolds, J. F., Treseder, K. K., & Wallenstein, M. D. (2008). Thermal adaptation of soil microbial respiration to elevated temperature. *Ecology Letters*, *11*(12), 1316–1327. https://doi.org/10.1111/j.1461-0248.2008.01251.x

Byrne, B., Baker, D. F., Basu, S., Bertolacci, M., Bowman, K. W., Carroll, D., Chatterjee, A., Chevallier, F., Ciais, P., Cressie, N., Crisp, D., Crowell, S., Deng, F., Deng, Z., Deutscher, N. M., Dubey, M., Feng, S., García, O., Griffith, D. W. T., Herkommer, B., Hu, L., Jacobson, A. R., Janardanan, R., Jeong, S., Johnson, M. S., Jones, D. B. A., Kivi, R., Liu, J., Liu, Z., Maksyutov, S., Miller, J. B., Miller, S. M., Morino, I., Notholt, J., Oda, T., O'Dell, C. W., Oh, Y.-S., Ohyama, H., Patra, P. K., Peiro, H., Petri, C., Philip, S., Pollard, D. F., Poulter, B., Remaud, M., Schuh, A., Sha, M. K., Shiomi, K., Strong, K., Sweeney, C., Té, Y., Tian, H., Velazco, V. A., Vrekoussis, M., Warneke, T., Worden, J. R., Wunch, D., Yao, Y., Yun, J., Zammit-Mangion, A., & Zeng, N. (2022). Pilot top-down CO_2 budget constrained by the v10 OCO-2 MIP version 1. https://doi.org/10.48588/npf6-sw92

Byrne, B., Baker, D. F., Basu, S., Bertolacci, M., Bowman, K. W., Carroll, D., Chatterjee, A., Chevallier, F., Ciais, P., Cressie, N., Crisp, D., Crowell, S., Deng, F., Deng, Z., Deutscher, N. M., Dubey, M. K., Feng, S., García, O. E., Griffith, D. W. T., Herkommer, B., Hu, L., Jacobson, A. R., Janardanan, R., Jeong, S., Johnson, M. S., Jones, D. B. A., Kivi, R., Liu, J., Liu, Z., Maksyutov, S., Miller, J. B., Miller, S. M., Morino, I., Notholt, J., Oda, T., O'Dell, C. W., Oh, Y.-S., Ohyama, H., Patra, P. K., Peiro, H., Petri, C., Philip, S., Pollard, D. F., Poulter, B., Remaud, M., Schuh, A., Sha, M. K., Shiomi, K., Strong, K., Sweeney, C., Té, Y., Tian, H., Velazco, V. A., Vrekoussis, M., Warneke, T., Worden, J. R., Wunch, D., Yao, Y., Yun, J., Zammit-Mangion, A., & Zeng, N. (2023). National CO_2 budgets (2015–2020) inferred from atmospheric CO_2

observations in support of the global stocktake. *Earth System Science Data*, *15*(2), 963–1004. https://doi.org/10.5194/essd-15-963-2023

Ciais, P., Yao, Y., Gasser, T., Baccini, A., Wang, Y., Lauerwald, R., Peng, S., Bastos, A., Li, W., Raymond, P. A., Canadell, J. G., Peters, G. P., Andres, R. J., Chang, J., Yue, C., Dolman, A. J., Haverd, V., Hartmann, J., Laruelle, G., Konings, A. G., King, A. W., Liu, Y., Luyssaert, S., Maignan, F., Patra, P. K., Peregon, A., Regnier, P., Pongratz, J., Poulter, B., Shvidenko, A., Valentini, R., Wang, R., Broquet, G., Yin, Y., Zscheischler, J., Guenet, B., Goll, D. S., Ballantyne, A. P., Yang, H., Qiu, C., & Zhu, D. (2021). Empirical estimates of regional carbon budgets imply reduced global soil heterotrophic respiration. *National Science Review*, *8*(2). https://doi.org/10.1093/nsr/nwaa145

Clark, D. B., Mercado, L. M., Sitch, S., Jones, C. D., Gedney, N., Best, M. J., Pryor, M., Rooney, G. G., Essery, R. L. H., Blyth, E., Boucher, O., Harding, R. J., Huntingford, C., & Cox, P. M. (2011). The Joint UK Land Environment Simulator (JULES), model description – part 2: Carbon fluxes and vegetation dynamics. *Geoscientific Model Development*, *4*(3), 701–722. https://doi.org/10.5194/gmd-4-701-2011

El Masri, B., Shu, S., & Jain, A. K. (2015). Implementation of a dynamic rooting depth and phenology into a land surface model: Evaluation of carbon, water, and energy fluxes in the high latitude ecosystems. *Agricultural and Forest Meteorology*, *211-212*, 85–99. https://doi.org/10.1016/j.agrformet.2015.06.002

Guimberteau, M., Zhu, D., Maignan, F., Huang, Y., Yue, C., Dantec-Nédélec, S., Ottlé, C., Jornet-Puig, A., Bastos, A., Laurent, P., Goll, D., Bowring, S., Chang, J., Guenet, B., Tifafi, M., Peng, S., Krinner, G., Ducharne, A., Wang, F., Wang, T., Wang, X., Wang, Y., Yin, Z., Lauerwald, R., Joetzjer, E., Qiu, C., Kim, H., & Ciais, P. (2018). ORCHIDEE-MICT (v8.4.1), a land surface model for the high latitudes: Model description and validation. *Geoscientific Model Development*, *11*(1), 121–163. https://doi.org/10.5194/gmd-11-121-2018

Huang, S., Arain, M. A., Arora, V. K., Yuan, F., Brodeur, J., & Peichl, M. (2011). Analysis of nitrogen controls on carbon and water exchanges in a conifer forest using the CLASS-CTEM^{N+} model. *Ecological Modelling*, *222*(20-22), 3743–3760. https://doi.org/10.1016/j.ecolmodel.2011.09.008

Huntzinger, D. N., Schwalm, C., Michalak, A. M., Schaefer, K., King, A. W., Wei, Y., Jacobson, A., Liu, S., Cook, R. B., Post, W. M., Berthier, G., Hayes, D., Huang, M., Ito, A., Lei, H., Lu, C., Mao, J., Peng, C. H., Peng, S., Poulter, B., Riccuito, D., Shi, X., Tian, H., Wang, W., Zeng, N., Zhao, F., & Zhu, Q. (2013). The North American Carbon Program Multi-scale Synthesis and Terrestrial Model Intercomparison Project – Part 1: Overview and experimental design. *Geoscientific Model Development*, *6*(6), 2121–2133. https://doi.org/10.5194/gmd-6-2121-2013

Ito, A. (2010). Changing ecophysiological processes and carbon budget in east asian ecosystems under near-future changes in climate: Implications for long-term monitoring from a process-

based model. *Journal of Plant Research*, *123*(4), 577–588. https://doi.org/10.1007/s10265-009-0305-x

Jain, A. K., & Yang, X. (2005). Modeling the effects of two different land cover change data sets on the carbon stocks of plants and soils in concert with CO_2 and climate change. *Global Biogeochemical Cycles*, *19*(2), GB2015. https://doi.org/10.1029/2004gb002349

Jung, M., Schwalm, C., Migliavacca, M., Walther, S., Camps-Valls, G., Koirala, S., Anthoni, P., Besnard, S., Bodesheim, P., Carvalhais, N., Chevallier, F., Gans, F., Goll, D. S., Haverd, V., Köhler, P., Ichii, K., Jain, A. K., Liu, J., Lombardozzi, D., Nabel, J. E. M. S., Nelson, J. A., O'Sullivan, M., Pallandt, M., Papale, D., Peters, W., Pongratz, J., Rödenbeck, C., Sitch, S., Tramontana, G., Walker, A., Weber, U., & Reichstein, M. (2020). Scaling carbon fluxes from eddy covariance sites to globe: Synthesis and evaluation of the FLUXCOM approach. *Biogeosciences*, *17*(5), 1343–1365. https://doi.org/10.5194/bg-17-1343-2020

Jung, M., Reichstein, M., Schwalm, C. R., Huntingford, C., Sitch, S., Ahlström, A., Arneth, A., Camps-Valls, G., Ciais, P., Friedlingstein, P., Gans, F., Ichii, K., Jain, A. K., Kato, E., Papale, D., Poulter, B., Raduly, B., Rödenbeck, C., Tramontana, G., Viovy, N., Wang, Y.-P., Weber, U., Zaehle, S., & Zeng, N. (2017). Compensatory water effects link yearly global land CO_2 sink changes to temperature. *Nature*, *541*(7638), 516–520. https://doi.org/10.1038/nature20780

Koven, C. D., Hugelius, G., Lawrence, D. M., & Wieder, W. R. (2017). Higher climatological temperature sensitivity of soil carbon in cold than warm climates. *Nature Climate Change*, *7*(11), 817–822. https://doi.org/10.1038/nclimate3421

Kowalczyk, E. A., Wang, Y. P., Law, R. M., Davies, H. L., McGregor, J. L., & Abramowitz, G. (2006). *The CSIRO Atmosphere Biosphere Land Exchange (CABLE) model for use in climate models and as an offline model* (tech. rep. No. 013). CSIRO Marine and Atmospheric Research. Aspendale, Victoria, Australia. https://doi.org/10.4225/08/58615c6a9a51d

Krinner, G., Viovy, N., de Noblet-Ducoudré, N., Ogée, J., Polcher, J., Friedlingstein, P., Ciais, P., Sitch, S., & Prentice, I. C. (2005). A dynamic global vegetation model for studies of the coupled atmosphere-biosphere system. *Global Biogeochemical Cycles*, *19*(1), GB1015. https://doi.org/10.1029/2003GB002199

Lei, H., Huang, M., Leung, L. R., Yang, D., Shi, X., Mao, J., Hayes, D. J., Schwalm, C. R., Wei, Y., & Liu, S. (2014). Sensitivity of global terrestrial gross primary production to hydrologic states simulated by the Community Land Model using two runoff parameterizations. *Journal of Advances in Modeling Earth Systems*, *6*(3), 658–679. https://doi.org/10.1002/2013MS000252

Levy, P. E., Cannell, M. G. R., & Friend, A. D. (2004). Modelling the impact of future changes in climate, CO_2 concentration and land use on natural ecosystems and the terrestrial carbon sink. *Global Environmental Change*, *14*(1), 21–30. https://doi.org/10.1016/j.gloenvcha.2003.10.005

Mao, J., Thornton, P. E., Shi, X., Zhao, M., & Post, W. M. (2012). Remote sensing evaluation of CLM4 GPP for the period 2000–09. *Journal of Climate*, *25*(15), 5327–5342. https://doi.org/10.1175/JCLI-D-11-00401.1

McGuire, A. D., Hayes, D. J., Kicklighter, D. W., Manizza, M., Zhuang, Q., Chen, M., Follows, M. J., Gurney, K. R., Mcclelland, J. W., Melillo, J. M., Peterson, B. J., & Prinn, R. G. (2010). An analysis of the carbon balance of the Arctic basin from 1997 to 2006. *Tellus B: Chemical and Physical Meteorology*, *62*(5), 455–474. https://doi.org/10.1111/j.1600-0889.2010.00497.x

Mesinger, F., DiMego, G., Kalnay, E., Mitchell, K., Shafran, P. C., Ebisuzaki, W., Jović, D., Woollen, J., Rogers, E., Berbery, E. H., Ek, M. B., Fan, Y., Grumbine, R., Higgins, W., Li, H., Lin, Y., Manikin, G., Parrish, D., & Shi, W. (2006). North American Regional Reanalysis. *Bulletin of the American Meteorological Society*, *87*(3), 343–360. https://doi.org/10.1175/BAMS-87-3-343

Monteith, J. L. (1972). Solar radiation and productivity in tropical ecosystems. *The Journal of Applied Ecology*, *9*(3), 747–766. https://doi.org/10.2307/2401901

Oleson, K. W., Lawrence, D. M., Gordon B. Bonan, B. D., Huang, M., Koven, C. D., Levis, S., Li, F., Riley, W. J., Subin, Z. M., Swenson, S. C., Thornton, P. E., Bozbiyik, A., Fisher, R., Heald, C. L., Kluzek, E., Lamarque, J.-F., Lawrence, P. J., Leung, L. R., Lipscomb, W., Muszala, S., Ricciuto, D. M., Sacks, W., Sun, Y., Tang, J., & Yang, Z.-L. (2013). *Technical description of version 4.5 of the Community Land Model (CLM)* (tech. rep. NCAR/TN-503+STR). National Center for Atmospheric Research. Boulder, Colorado, USA. https://doi.org/10.5065/D6RR1W7M

Reichstein, M., Tenhunen, J. D., Roupsard, O., Ourcival, J.-M., Rambal, S., Dore, S., & Valentini, R. (2002). Ecosystem respiration in two Mediterranean evergreen holm oak forests: Drought effects and decomposition dynamics. *Functional Ecology*, *16*(1), 27–39. https://doi.org/10.1046/j.0269-8463.2001.00597.x

Ricciuto, D. M., King, A. W., Dragoni, D., & Post, W. M. (2011). Parameter and prediction uncertainty in an optimized terrestrial carbon cycle model: Effects of constraining variables and data record length. *Journal of Geophysical Research*, *116*(G1), G01033. https://doi.org/10.1029/2010jg001400

Schaefer, K., Collatz, G. J., Tans, P., Denning, A. S., Baker, I., Berry, J., Prihodko, L., Suits, N., & Philpott, A. (2008). Combined Simple Biosphere/Carnegie-Ames-Stanford Approach terrestrial carbon cycle model. *Journal of Geophysical Research*, *113*(G3), G03034. https://doi.org/10.1029/2007jg000603

Shiga, Y. P., Tadić, J. M., Qiu, X., Yadav, V., Andrews, A. E., Berry, J. A., & Michalak, A. M. (2018). Atmospheric CO_2 observations reveal strong correlation between regional net biospheric carbon uptake and solar-induced chlorophyll fluorescence. *Geophysical Research Letters*, *45*(2), 1122–1132. https://doi.org/10.1002/2017gl076630

Sitch, S., Smith, B., Prentice, I. C., Arneth, A., Bondeau, A., Cramer, W., Kaplan, J. O., Levis, S., Lucht, W., Sykes, M. T., Thonicke, K., & Venevsky, S. (2003). Evaluation of ecosystem dynamics, plant geography and terrestrial carbon cycling in the LPJ dynamic global vegetation model. *Global Change Biology*, *9*(2), 161–185. https://doi.org/10.1046/j.1365-2486.2003.00569.x

Stöckli, R., Lawrence, D. M., Niu, G.-Y., Oleson, K. W., Thornton, P. E., Yang, Z.-L., Bonan, G. B., Denning, A. S., & Running, S. W. (2008). Use of FLUXNET in the community land model development. *Journal of Geophysical Research: Biogeosciences*, *113*(G1). https://doi.org/10.1029/2007JG000562

Sun, W., Fang, Y., Luo, X., Shiga, Y. P., Zhang, Y., Andrews, A. E., Thoning, K. W., Fisher, J. B., Keenan, T. F., & Michalak, A. M. (2021). Midwest US croplands determine model divergence in North American carbon fluxes. *AGU Advances*, *2*(2), e2020AV000310. https://doi.org/10.1029/2020AV000310

Thornton, P. E., Law, B. E., Gholz, H. L., Clark, K. L., Falge, E., Ellsworth, D. S., Goldstein, A. H., Monson, R. K., Hollinger, D., Falk, M., Chen, J., & Sparks, J. P. (2002). Modeling and measuring the effects of disturbance history and climate on carbon and water budgets in evergreen needleleaf forests. *Agricultural and Forest Meteorology*, *113*(1-4), 185–222. https://doi.org/10.1016/s0168-1923(02)00108-9

Tian, H., Chen, G., Zhang, C., Liu, M., Sun, G., Chappelka, A., Ren, W., Xu, X., Lu, C., Pan, S., Chen, H., Hui, D., McNulty, S., Lockaby, G., & Vance, E. (2012). Century-scale responses of ecosystem carbon storage and flux to multiple environmental changes in the southern United States. *Ecosystems*, *15*(4), 674–694. https://doi.org/10.1007/s10021-012-9539-x

Woodward, F. I., Smith, T. M., & Emanuel, W. R. (1995). A global land primary productivity and phytogeography model. *Global Biogeochemical Cycles*, *9*(4), 471–490. https://doi.org/10.1029/95gb02432

Zaehle, S., & Friend, A. D. (2010). Carbon and nitrogen cycle dynamics in the O-CN land surface model: 1. model description, site-scale evaluation, and sensitivity to parameter estimates. *Global Biogeochemical Cycles*, *24*(1), GB1005. https://doi.org/10.1029/2009gb003521

Zeng, N., Mariotti, A., & Wetzel, P. (2005). Terrestrial mechanisms of interannual CO_2 variability. *Global Biogeochemical Cycles*, *19*(1), GB1016. https://doi.org/10.1029/2004GB002273

Zeng, N., Zhao, F., Collatz, G. J., Kalnay, E., Salawitch, R. J., West, T. O., & Guanter, L. (2014). Agricultural green revolution as a driver of increasing atmospheric CO_2 seasonal amplitude. *Nature*, *515*(7527), 394–397. https://doi.org/10.1038/nature13893

Zhu, Q., Liu, J., Peng, C., Chen, H., Fang, X., Jiang, H., Yang, G., Zhu, D., Wang, W., & Zhou, X. (2014). Modelling methane emissions from natural wetlands by development and application of the TRIPLEX-GHG model. *Geoscientific Model Development*, *7*(3), 981–999. https://doi.org/10.5194/gmd-7-981-2014
