# Supplementary material for: Biome-scale temperature sensitivity of ecosystem respiration revealed by atmospheric CO2 observations
Source: Nat Ecol Evol. 2023 Jun 15;7(8):1199–210. doi: 10.1038/s41559-023-02093-x (PMC10406605; doi:10.1038/s41559-023-02093-x)
Supplement: Supplementary file 2 — Reporting Summary [file 41559_2023_2093_MOESM2_ESM.pdf]

nature portfolio

Corresponding author(s): Wu Sun
Anna M. Michalak

Last updated by author(s): Apr 28, 2023

Reporting Summary

Nature Portfolio wishes to improve the reproducibility of the work that we publish. This form provides structure for consistency and transparency in reporting. For further information on Nature Portfolio policies, see our Editorial Policies and the Editorial Policy Checklist.

Statistics

For all statistical analyses, confirm that the following items are present in the figure legend, table legend, main text, or Methods section.

n/a	Confirmed	
☐	☒	The exact sample size (*n*) for each experimental group/condition, given as a discrete number and unit of measurement
☒	☐	A statement on whether measurements were taken from distinct samples or whether the same sample was measured repeatedly
☐	☒	The statistical test(s) used AND whether they are one- or two-sided<br>*Only common tests should be described solely by name; describe more complex techniques in the Methods section.*
☐	☒	A description of all covariates tested
☐	☒	A description of any assumptions or corrections, such as tests of normality and adjustment for multiple comparisons
☐	☒	A full description of the statistical parameters including central tendency (e.g. means) or other basic estimates (e.g. regression coefficient) AND variation (e.g. standard deviation) or associated estimates of uncertainty (e.g. confidence intervals)
☒	☐	For null hypothesis testing, the test statistic (e.g. *F*, *t*, *r*) with confidence intervals, effect sizes, degrees of freedom and *P* value noted<br>*Give P values as exact values whenever suitable.*
☒	☐	For Bayesian analysis, information on the choice of priors and Markov chain Monte Carlo settings
☒	☐	For hierarchical and complex designs, identification of the appropriate level for tests and full reporting of outcomes
☐	☒	Estimates of effect sizes (e.g. Cohen's *d*, Pearson's *r*), indicating how they were calculated

Our web collection on statistics for biologists contains articles on many of the points above.

Software and code

Policy information about availability of computer code

Data collection

We did not use software in data collection. Publicly available data were provided by individual PIs.

Data analysis

Code used to generate figures and results is archived at https://doi.org/10.5281/zenodo.7874439.
The following software environment is required to run the code:
Python >= 3.8 (Python 2 not supported)
jupyterlab >= 3.2.1
numpy >= 1.21.2
scipy >= 1.7.2
pandas >= 1.3.4
xarray >= 0.19.0
statsmodels >= 0.13.0
matplotlib >= 3.5.0
cartopy >= 0.18.0
seaborn >= 0.11.2

For manuscripts utilizing custom algorithms or software that are central to the research but not yet described in published literature, software must be made available to editors and reviewers. We strongly encourage code deposition in a community repository (e.g. GitHub). See the Nature Portfolio guidelines for submitting code & software for further information.

Data

Policy information about availability of data

All manuscripts must include a data availability statement. This statement should provide the following information, where applicable:

- Accession codes, unique identifiers, or web links for publicly available datasets
- A description of any restrictions on data availability
- For clinical datasets or third party data, please ensure that the statement adheres to our policy

Results presented in this study are available at https://doi.org/10.5281/zenodo.7874439. The ObsPack GLOBALVIEWplus CO2 data product is available at https://www.esrl.noaa.gov/gmd/ccgg/obspack. The CarbonTracker-Lagrange WRF-STILT footprints are available at https://www.esrl.noaa.gov/gmd/ccgg/carbontracker-lagrange/. The FFDAS v2 data product is available at https://ffdas.rc.nau.edu/. The North American Regional Reanalysis data can be obtained at https://psl.noaa.gov/data/gridded/data.narr.html. The MsTMIP v2 model ensemble is available at https://nacp.ornl.gov/. The TRENDY v6 model ensemble is available at https://sites.exeter.ac.uk/trendy/. The FLUXCOM model ensemble is available at http://www.fluxcom.org/.

Human research participants

Policy information about studies involving human research participants and Sex and Gender in Research.

Reporting on sex and gender	N/A
Population characteristics	N/A
Recruitment	N/A
Ethics oversight	N/A

Note that full information on the approval of the study protocol must also be provided in the manuscript.

Field-specific reporting

Please select the one below that is the best fit for your research. If you are not sure, read the appropriate sections before making your selection.

☐ Life sciences ☐ Behavioural & social sciences ☒ Ecological, evolutionary & environmental sciences

For a reference copy of the document with all sections, see nature.com/documents/nr-reporting-summary-flat.pdf

Ecological, evolutionary & environmental sciences study design

All studies must disclose on these points even when the disclosure is negative.

Study description	We use observations of atmospheric CO2 concentrations from a network of towers together with carbon flux estimates from state-of-the-art terrestrial biosphere models to characterize the large-scale temperature sensitivity of ecosystem respiration for various North American biomes.
Research sample	39,217 observations of atmospheric CO2 concentration from a network of 44 towers over North America during 2007–2010;<br>29 terrestrial biosphere model simulations from 24 independent models;<br>3 data-driven models for carbon flux estimates in the FLUXCOM ensemble
Sampling strategy	We used all available data after filtering and quality control.
Data collection	Atmospheric CO2 concentrations were collected by individual site PIs across North America. The WRF-STILT transport footprints were provided by the NOAA CarbonTracker-Lagrange Team. Terrestrial biosphere model and data-driven model simulations were provided by individual modelers.
Timing and spatial scale	The study domain covers the North American continent and individual biomes therein. Atmospheric CO2 concentration data were reported at three-hourly time scale from 44 sites across North America, spanning the period 2007–2010. The WRF-STILT transport footprints were at three-hourly time scale and 1° × 1° spatial resolution. Carbon flux estimates from terrestrial biosphere models were at the monthly time scale. All carbon flux estimates from terrestrial biosphere models were harmonized at 1° × 1° spatial resolution.
Data exclusions	We used all relevant data to derive estimates of temperature sensitivity for individual models. For the derivation of an overall temperature sensitivity across models, three terrestrial biosphere models were excluded due to low explanatory power of their estimates of gross primary productivity. This practice has been documented in the methods and reporting.
Reproducibility	Uncertainty ranges of estimates were reported. We have also released the code used to perform the analysis in a publicly accessible

Reproducibility	repository.
Randomization	Randomization is not relevant as we examined the effect over the entire study domain.
Blinding	Blind is not relevant because the work was not experimental in nature.

Did the study involve field work? ☐ Yes ☒ No

Reporting for specific materials, systems and methods

We require information from authors about some types of materials, experimental systems and methods used in many studies. Here, indicate whether each material, system or method listed is relevant to your study. If you are not sure if a list item applies to your research, read the appropriate section before selecting a response.

Materials & experimental systems

n/a	Involved in the study
☒	☐ Antibodies
☒	☐ Eukaryotic cell lines
☒	☐ Palaeontology and archaeology
☒	☐ Animals and other organisms
☒	☐ Clinical data
☒	☐ Dual use research of concern

Methods

n/a	Involved in the study
☒	☐ ChIP-seq
☒	☐ Flow cytometry
☒	☐ MRI-based neuroimaging
